# Ovarian cancer pathology characteristics as predictors of variant pathogenicity in *BRCA1* and *BRCA2*

**DOI:** 10.1038/s41416-023-02263-5

**Published:** 2023-04-19

**Authors:** Denise G. O’Mahony, Susan J. Ramus, Melissa C. Southey, Nicola S. Meagher, Andreas Hadjisavvas, Esther M. John, Ute Hamann, Evgeny N. Imyanitov, Irene L. Andrulis, Priyanka Sharma, Mary B. Daly, Christopher R. Hake, Jeffrey N. Weitzel, Anna Jakubowska, Andrew K. Godwin, Adalgeir Arason, Anita Bane, Jacques Simard, Penny Soucy, Maria A. Caligo, Phuong L. Mai, Kathleen B. M. Claes, Manuel R. Teixeira, Wendy K. Chung, Conxi Lazaro, Peter J. Hulick, Amanda E. Toland, Inge Sokilde Pedersen, HEBON Investigators, Marian J. E. Mourits, Susan L. Neuhausen, Ana Vega, Miguel de la Hoya, Heli Nevanlinna, Mallika Dhawan, Valentina Zampiga, Rita Danesi, Liliana Varesco, Viviana Gismondi, Valerio Gaetano Vellone, Paul A. James, Ramunas Janavicius, Liene Nikitina-Zake, Finn Cilius Nielsen, Thomas van Overeem Hansen, Tanja Pejovic, Ake Borg, Johanna Rantala, Kenneth Offit, Marco Montagna, Katherine L. Nathanson, Susan M. Domchek, Ana Osorio, María J. García, Beth Y. Karlan, GEMO Study Collaborators, Fabienne Lesueur, Anna De Fazio, David Bowtell, AOCS Group, Anna De Fazio, Lesley McGuffog, Goska Leslie, Michael T. Parsons, Thilo Dörk, Lisa-Marie Speith, Elizabeth Santana dos Santos, Alexandre André B. A. da Costa, Paolo Radice, Paolo Peterlongo, Laura Papi, Christoph Engel, Eric Hahnen, Rita K. Schmutzler, Barbara Wappenschmidt, Douglas F. Easton, Marc Tischkowitz, Christian F. Singer, Yen Yen Tan, Alice S. Whittemore, Weiva Sieh, James D. Brenton, Drakoulis Yannoukakos, Florentia Fostira, Irene Konstantopoulou, Jana Soukupova, Michal Vocka, CZECANCA Consortium, Georgia Chenevix-Trench, Paul D. P. Pharoah, Antonis C. Antoniou, David E. Goldgar, Amanda B. Spurdle, Kyriaki Michailidou, The Consortium of Investigators of Modifiers of BRCA1/2, Miguel de la Hoya, Thomas van Overeem Hansen, Elizabeth Santana dos Santos, Evidence-based Network for the Interpretation of Germline Mutant Alleles Consortium

**Affiliations:** 1 Biostatistics Unit, The Cyprus Institute of Neurology and Genetics, Nicosia, 2371 Cyprus; 2 Department of Cancer Genetics, Therapeutics and Ultrastructural Pathology, The Cyprus Institute of Neurology and Genetics, Nicosia, 2371 Cyprus; 3 School of Clinical Medicine, University of New South Wales Medicine and Health, University of New South Wales Sydney, Sydney, NSW 2052 Australia; 4 Adult Cancer Program, Lowy Cancer Research Centre, University of New South Wales Sydney, Sydney, NSW 2052 Australia; 5 Precision Medicine, School of Clinical Sciences at Monash Health, Monash University, Clayton, VIC 3168 Australia; 6 Department of Clinical Pathology, The University of Melbourne, Melbourne, VIC 3010 Australia; 7 Cancer Epidemiology Division, Cancer Council Victoria, Melbourne, VIC 3004 Australia; 8 The Daffodil Centre, The University of Sydney, a joint venture with Cancer Council NSW, Sydney, NSW Australia; 9 Department of Epidemiology and Population Health, Stanford University School of Medicine, Stanford, CA 94305 USA; 10 Department of Medicine, Division of Oncology, Stanford Cancer Institute, Stanford University School of Medicine, Stanford, CA 94304 USA; 11 Molecular Genetics of Breast Cancer, German Cancer Research Center (DKFZ), Heidelberg, 69120 Germany; 12 N.N. Petrov Institute of Oncology, St. Petersburg, 197758 Russia; 13 Fred A. Litwin Center for Cancer Genetics, Lunenfeld-Tanenbaum Research Institute of Mount Sinai Hospital, Toronto, ON M5G 1×5 Canada; 14 Department of Molecular Genetics, University of Toronto, Toronto, ON M5S 1A8 Canada; 15 Department of Internal Medicine, Division of Medical Oncology, University of Kansas Medical Center, Westwood, KS 66205 USA; 16 Department of Clinical Genetics, Fox Chase Cancer Center, Philadelphia, PA 19111 USA; 17 Waukesha Memorial Hospital-Pro Health Care, Waukesha, WI 53188 USA; 18 The University of Kansas Cancer Center, Kansas City, MO 66160 USA; 19 Department of Genetics and Pathology, International Hereditary Cancer Center, Pomeranian Medical University, Szczecin, 171-252 Poland; 20 Independent Laboratory of Molecular Biology and Genetic Diagnostics, Pomeranian Medical University, Szczecin, 171-252 Poland; 21 Department of Pathology and Laboratory Medicine, The University of Kansas Medical Center, Kansas City, KS 66160 USA; 22 Department of Pathology, Landspitali University Hospital, Reykjavik, 101 Iceland; 23 BMC (Biomedical Centre), Faculty of Medicine, University of Iceland, Reykjavik, 101 Iceland; 24 Department of Pathology & Molecular Medicine, Juravinski Hospital and Cancer Centre, McMaster University, Hamilton, ON L8V 1C3 Canada; 25 Genomics Center, Centre Hospitalier Universitaire de Québec – Université Laval Research Center, Québec City, QC G1V 4G2 Canada; 26 SOD Genetica Molecolare, University Hospital, Pisa, 56126 Italy; 27 Magee-Womens Hospital, University of Pittsburgh School of Medicine, Pittsburgh, PA 15213 USA; 28 Centre for Medical Genetics, Ghent University, Gent, 9000 Belgium; 29 Department of Laboratory Genetics, Portuguese Oncology Institute of Porto (IPO Porto)/Comprehensive Cancer Center, Porto, 4200-072 Portugal; 30 School of Medicine and Biomedical Sciences Institute (ICBAS), University of Porto, Porto, 4050-013 Portugal; 31 Departments of Pediatrics and Medicine, Columbia University, New York, NY 10032 USA; 32 Hereditary Cancer Program, ONCOBELL-IDIBELL-IGTP, Catalan Institute of Oncology, CIBERONC, Barcelona, 08908 Spain; 33 Center for Medical Genetics, NorthShore University HealthSystem, Evanston, IL 60201 USA; 34 The University of Chicago Pritzker School of Medicine, Chicago, IL 60637 USA; 35 Department of Cancer Biology and Genetics, The Ohio State University, Columbus, OH 43210 USA; 36 Molecular Diagnostics, Aalborg University Hospital, Aalborg, 9000 Denmark; 37 Clinical Cancer Research Center, Aalborg University Hospital, Aalborg, 9000 Denmark; 38 Department of Clinical Medicine, Aalborg University, Aalborg, 9000 Denmark; 39 Department of Population Sciences, Beckman Research Institute of City of Hope, Duarte, CA 91010 USA; 40 Centro de Investigación en Red de Enfermedades Raras (CIBERER), Madrid, 28029 Spain; 41 Fundación Pública Galega de Medicina Xenómica, Santiago de Compostela, 15706 Spain; 42 Instituto de Investigación Sanitaria de Santiago de Compostela (IDIS), Complejo Hospitalario Universitario de Santiago, SERGAS, Santiago de Compostela, 15706 Spain; 43 Molecular Oncology Laboratory, CIBERONC, Hospital Clinico San Carlos, IdISSC (Instituto de Investigación Sanitaria del Hospital Clínico San Carlos), Madrid, 28040 Spain; 44 Department of Obstetrics and Gynecology, Helsinki University Hospital, University of Helsinki, Helsinki, 00290 Finland; 45 Cancer Genetics and Prevention Program, University of California San Francisco, San Francisco, CA 94143-1714 USA; 46 Biosciences Laboratory, IRCCS Istituto Romagnolo per lo Studio dei Tumori (IRST) “Dino Amadori”, Meldola, Italy; 47 Romagna Cancer Registry, IRCCS Istituto Romagnolo per lo Studio dei Tumori (IRST) “Dino Amadori”, Meldola, 47014 Italy; 48 Unit of Hereditary Cancer, IRCCS Ospedale Policlinico San Martino, Genoa, 16132 Italy; 49 Unit of Pathology, IRCCS Ospedale Policlinico San Martino, Genoa, 16132 Italy; 50 Department of Gynecology and Obstetrics, Ribeirao Preto Medical School, University of Sao Paulo, Ribeirao Preto, 14049-900 Brazil; 51 Faculty of Medicine, Institute of Biomedical Sciences, Department of Human and Medical Genetics, Vilnius University, Vilnius, LT-03101 Lithuania; 52 State Research Institute Centre for Innovative Medicine, Vilnius, 8410 Lithuania; 53 Latvian Biomedical Research and Study Centre, Riga, LV-1067 Latvia; 54 Center for Genomic Medicine, Rigshospitalet, Copenhagen University Hospital, Copenhagen, DK-2100 Denmark; 55 Department of Clinical Genetics, Rigshospitalet, Copenhagen University Hospital, Copenhagen, DK-2100 Denmark; 56 Department of Clinical Medicine, Faculty of Health and Medical Sciences, , University of Copenhagen, Copenhagen, 2200 Denmark; 57 Department of Obstetrics and Gynecology, Oregon Health & Science University, Portland, OR 97239 USA; 58 Knight Cancer Institute, Oregon Health & Science University, Portland, OR 97239 USA; 59 Department of Oncology, Lund University and Skåne University Hospital, Lund, 222 41 Sweden; 60 Clinical Genetics, Karolinska Institutet, Stockholm, 171 76 Sweden; 61 Clinical Genetics Research Lab, Department of Cancer Biology and Genetics, Memorial Sloan Kettering Cancer Center, New York, NY 10065 USA; 62 Clinical Genetics Service, Department of Medicine, Memorial Sloan Kettering Cancer Center, New York, NY 10065 USA; 63 Immunology and Molecular Oncology Unit, Veneto Institute of Oncology IOV - IRCCS, Padua, 35128 Italy; 64 Basser Center for BRCA, Abramson Cancer Center, University of Pennsylvania, Philadelphia, PA 19066 USA; 65 Human Genetics Group, Spanish National Cancer Research Centre (CNIO), Madrid, 28029 Spain; 66 Centre for Biomedical Network Research on Rare Diseases (CIBERER), Instituto de Salud Carlos III, Madrid, 28029 Spain; 67 Genetics Service, Fundación Jiménez Díaz, Madrid, 28040 Spain; 68 Computational Oncology Group, Structural Biology Programme, Spanish National Cancer Research Centre (CNIO), Madrid, 28029 Spain; 69 David Geffen School of Medicine, Department of Obstetrics and Gynecology, University of California at Los Angeles, Los Angeles, CA 90095 USA; 70 Centre for Cancer Research, The Westmead Institute for Medical Research, Sydney, Australia; 71 Department of Gynaecological Oncology, Westmead Hospital, Sydney, NSW 2145 Australia; 72 The University of Sydney, Sydney, NSW 2145 Australia; 73 Peter MacCallum Cancer Centre, Melbourne, VIC 3000 Australia; 74 Sir Peter MacCallum Department of Oncology, The University of Melbourne, Melbourne, VIC 3000 Australia; 75 Centre for Cancer Genetic Epidemiology, Department of Public Health and Primary Care, University of Cambridge, Cambridge, CB1 8RN UK; 76 Population Health Program, QIMR Berghofer Medical Research Institute, Brisbane, QLD 4006 Australia; 77 Gynaecology Research Unit, Hannover Medical School, Hannover, 30625 Germany; 78 Service de Génétique, Institut Curie, Paris, 75005 France; 79 Oncology Center, Hospital Sirio-Libanes, São Paulo, 01308-050 Brazil; 80 Department of Clinical Oncology, A.C.Camargo Cancer Center, São Paulo, 1509900 Brazil; 81 Department of Radiation Oncology, Dana-Farber Cancer Institute, Boston, MA 2215 USA; 82 Unit of Preventive Medicine: Molecular Bases of Genetic Risk, Department of Experimental Oncology, Fondazione IRCCS Istituto Nazionale dei Tumori (INT), Milan, 20133 Italy; 83 Genome Diagnostics Program, IFOM ETS - the AIRC Institute of Molecular Oncology, Milan, 20139 Italy; 84 Department of Experimental and Clinical Biomedical Sciences ‘Mario Serio’, Medical Genetics Unit, University of Florence, Florence, 27571 Italy; 85 Institute for Medical Informatics, Statistics and Epidemiology, University of Leipzig, Leipzig, 04107 Germany; 86 LIFE - Leipzig Research Centre for Civilization Diseases, University of Leipzig, Leipzig, 04103 Germany; 87 Center for Familial Breast and Ovarian Cancer, Faculty of Medicine and University Hospital Cologne, University of Cologne, Cologne, 50937 Germany; 88 Center for Integrated Oncology (CIO), Faculty of Medicine and University Hospital Cologne, University of Cologne, Cologne, 50937 Germany; 89 Center for Molecular Medicine Cologne (CMMC), Faculty of Medicine and University Hospital Cologne, University of Cologne, Cologne, 50931 Germany; 90 Centre for Cancer Genetic Epidemiology, Department of Oncology, University of Cambridge, Cambridge, CB1 8RN UK; 91 Program in Cancer Genetics, Departments of Human Genetics and Oncology, McGill University, Montréal, QC H4A 3J1 Canada; 92 Department of Medical Genetics, National Institute for Health Research Cambridge Biomedical Research Centre, University of Cambridge, Cambridge, CB2 0QQ UK; 93 Department of OB/GYN and Comprehensive Cancer Center, Medical University of Vienna, Vienna, 1090 Austria; 94 Department of Biomedical Data Science, Stanford University School of Medicine, Stanford, CA 94305 USA; 95 Department of Population Health Science and Policy, Icahn School of Medicine at Mount Sinai, New York, NY 10029 USA; 96 Department of Genetics and Genomic Sciences, Icahn School of Medicine at Mount Sinai, New York, NY 10029 USA; 97 Cancer Research UK Cambridge Institute, University of Cambridge, Cambridge, CB2 0RE UK; 98 Molecular Diagnostics Laboratory, INRASTES, National Centre for Scientific Research ‘Demokritos’, Athens, 15310 Greece; 99 Institute of Medical Biochemistry and Laboratory Diagnostics, First Faculty of Medicine, Charles University and General University Hospital in Prague, Prague, 12000 Czech Republic; 100 Department of Oncology, First Faculty of Medicine, Charles University and General University Hospital in Prague, Prague, 12000 Czech Republic; 101 Department of Genetics and Computational Biology, QIMR Berghofer Medical Research Institute, Brisbane, QLD 4006 Australia; 102 Department of Dermatology, Huntsman Cancer Institute, University of Utah School of Medicine, Salt Lake City, UT 84112 USA; 103 Department of Obstetrics and Gynecology, University Medical Center Groningen, University Groningen, Groningen, 9713 GZ the Netherlands; 104 The Hereditary Breast and Ovarian Cancer Research Group Netherlands (HEBON), Coordinating Center: The Netherlands Cancer Institute, Amsterdam, 1066 CX the Netherlands; 105 Genetic Epidemiology of Cancer team, Inserm U900, Paris, 75005 France; 106 Institut Curie, Paris, 75005 France; 107 Mines ParisTech, Fontainebleau, 77305 France

**Keywords:** Breast cancer, Genetic testing, Breast cancer, Epidemiology

## Abstract

**Background:**

The distribution of ovarian tumour characteristics differs between germline *BRCA1* and *BRCA2* pathogenic variant carriers and non-carriers. In this study, we assessed the utility of ovarian tumour characteristics as predictors of *BRCA1* and *BRCA2* variant pathogenicity, for application using the American College of Medical Genetics and the Association for Molecular Pathology (ACMG/AMP) variant classification system.

**Methods:**

Data for 10,373 ovarian cancer cases, including carriers and non-carriers of *BRCA1* or *BRCA2* pathogenic variants, were collected from unpublished international cohorts and consortia and published studies. Likelihood ratios (LR) were calculated for the association of ovarian cancer histology and other characteristics, with *BRCA1* and *BRCA2* variant pathogenicity. Estimates were aligned to ACMG/AMP code strengths (supporting, moderate, strong).

**Results:**

No histological subtype provided informative ACMG/AMP evidence in favour of *BRCA1* and *BRCA2* variant pathogenicity. Evidence against variant pathogenicity was estimated for the mucinous and clear cell histologies (supporting) and borderline cases (moderate). Refined associations are provided according to tumour grade, invasion and age at diagnosis.

**Conclusions:**

We provide detailed estimates for predicting *BRCA1* and *BRCA2* variant pathogenicity based on ovarian tumour characteristics. This evidence can be combined with other variant information under the ACMG/AMP classification system, to improve classification and carrier clinical management.

## Introduction

Ovarian cancer can be classified based on tumour origin into epithelial (~90% of all cases [1]), sex cord/stromal and germ cell. The epithelial cases differentiate into five main histological subtypes (“histotypes”), including high-grade serous carcinomas (HGSC), which is the most frequent subtype [2], low-grade serous carcinomas (LGSC), mucinous, endometrioid and clear cell ovarian cancers [3]. Rarer forms of epithelial ovarian cancer such as transitional cell or mesenchymal and mixed-epithelial carcinomas may also occur [3]. Due to their differences in morphological, molecular and clinical characteristics [4], ovarian cancer histotypes are considered different diseases [5].

Several genes have been associated with increased risk of ovarian cancer, including *BRCA1* and *BRCA2* [6]*, PALB2, BRIP1* [7], *RAD51C* and *RAD51D* [8], with the largest percentage of cases (10-15%) being attributable to germline pathogenic variants in *BRCA1* or *BRCA2* [9]. Previous findings have suggested that the distribution of ovarian cancer histopathology subtypes differs in germline *BRCA1* and *BRCA2* pathogenic variant carriers, compared to non-carriers, with a similar distribution associated with pathogenic variants for the two genes [10]. Germline pathogenic variants in the two genes occur predominantly in patients diagnosed with HGSC, where the probability of finding a *BRCA1* or *BRCA2* pathogenic variant reaches as high as 25.2% [11]. Identification of *BRCA1* and *BRCA2* pathogenic variants is lower for patients with endometrioid carcinomas (4.17–10.3% [12, 13]), LGSC (1.2–6.0% [14]) and clear cell carcinomas (2.8–9.1% [15, 16]). Earlier work also suggests that germline *BRCA1* and *BRCA2* pathogenic variants are unlikely to be found in patients with tumours of mucinous histology (0 to 4% [17–19]). Borderline tumours, a separate entity of non-invasive epithelial ovarian cancers, are also characterised by a low frequency of *BRCA1* and *BRCA2* germline pathogenic variants [10]. The National Comprehensive Cancer Network (NCCN) [20], American Society of Clinical Oncology (ASCO) [21] as well as others [22], recommend germline *BRCA1* and *BRCA2* genetic testing for all epithelial ovarian cancer patients irrespective of histology. Other national and international medical societies and panels suggest selective testing for HGSC or non-mucinous ovarian cancer histological subtypes, due to the higher probability of finding a *BRCA1* or *BRCA2* pathogenic variant [23, 24].

*BRCA1* and *BRCA2* genetic testing applied for the identification of high-risk individuals will often (5–10%) identify variants of uncertain significance (VUS) [25]. VUS are characterised by insufficient evidence for their association with disease pathogenicity and consequent clinical uncertainty in making informed decisions on disease management [26, 27]. It is recommended that VUS detection is not incorporated in patient risk assessment, and carriers are managed according to their clinical features and family history, which reduces the possibility of receiving risk-reducing interventions being offered to carriers of pathogenic variants [28, 29]. To facilitate VUS classification efforts, the American College of Medical Genetics and the Association for Molecular Pathology (ACMG/AMP) groups have developed standards and guidelines that are widely applied by clinical labs. This system weights independent lines of evidence for and against variant pathogenicity as ‘very strong’, ‘strong’, ‘moderate’ and ‘supporting’ [30, 31]. These strengths are combined based on a scoring system of criteria to classify variants. The evidence considered may include variant location, predicted coding effect, functional data, variant co-segregation with disease or variant frequency in affected and non-affected individuals. Recently, the model was transformed into a Bayesian framework, in which weights were aligned to pathogenic and benign Likelihood ratio (LR) evidence [32].

In addition, the Multifactorial Likelihood model, applied by the Evidence‐based Network for the Interpretation of Germline Mutant Alleles (ENIGMA) consortium, also has been used to weight different evidence types for *BRCA1* and *BRCA2* variant classification efforts [33, 34]. For a given variant, the model calculates the posterior probability of pathogenicity, in a Bayesian quantitative classification framework that integrates multiple independent lines of evidence for the association of a variant with pathogenicity, measured by LRs, with calibrated prior probabilities of pathogenicity determined through in silico predictions [35].

Despite the observed associations between *BRCA1* and *BRCA2* germline pathogenic variant status with ovarian tumour characteristics, currently VUS interpretation efforts do not consider ovarian tumour pathology. We performed analyses on a large collection of data from ovarian cancer cases, including *BRCA1* and *BRCA2* (likely) pathogenic variant carriers and non-carriers, to assess histology and other tumour characteristics as predictors of germline *BRCA1* and *BRCA2* variant pathogenicity, with the aim of standardising the application of this evidence in clinical variant curation using the ACMG/AMP classification system, to inform the future interpretation of VUS in *BRCA1* and *BRCA2*.

## Materials and methods

### Data collection and selection criteria

**Fig. 1:**
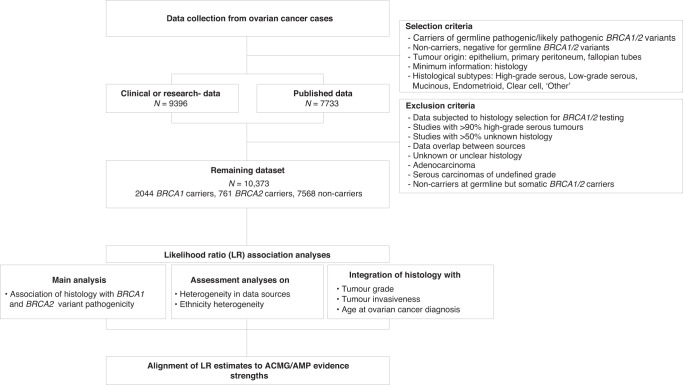
Flowchart of study design and methods. Carriers refer to individuals with a reported germline pathogenic or likely pathogenic variant in *BRCA1* or *BRCA2*. Non-carriers refer to individuals tested negative for *BRCA1* and/or *BRCA2* in germline. The ‘other’ category comprised rare forms of ovarian cancer not belonging to any of the other subtypes, including tumours defined as: ‘other’ by data sources not specifying tumour histology; mixed-epithelial carcinomas; carcinosarcomas, transitional cell (Brenner tumours), undifferentiated or poorly differentiated; squamous cell. HGSC high-grade serous carcinoma, LGSC low-grade serous carcinoma.

An overview of the data collection process is shown in Fig. 1, where the selection and exclusion criteria are stated. In this study, data from ovarian cancer cases were collected (ovarian epithelium, primary peritoneum or fallopian tubes as primary sites) from reported germline *BRCA1* and *BRCA2* pathogenic or likely pathogenic variant carriers and individuals who tested negative for germline *BRCA1* and *BRCA2* (likely) pathogenic variants (non-carriers), with known histology information. Variant class (pathogenic or likely pathogenic) was based on the classification assigned by contributing sources at the time of collection. The main tumour information analysed was ovarian tumour histology, where the histological subtypes ('histotypes') considered were in accordance with the most recent ovarian tumour classification system defined by the World Health Organisation (WHO) [3]. Only data falling into these histological categories were considered. These included: high-grade serous carcinomas (HGSC), low-grade serous carcinomas (LGSC), mucinous carcinomas, endometrioid carcinomas, clear cell carcinomas and the ‘other’ category. The ‘other’ category comprised rare forms of ovarian cancer not belonging to the above-mentioned subtypes and included tumours defined as: ‘other’ by data sources not specifying tumour histology; mixed-epithelial carcinomas; carcinosarcomas; transitional cell carcinomas (Brenner tumours); undifferentiated or poorly differentiated carcinomas; squamous cell carcinomas.

Data sources included clinically- or research-tested data, as well as data from published studies. Specifically, we initially collected data for 9396 individuals from clinical or research sources, subjected to germline *BRCA1* and *BRCA2* genetic testing, from the CIMBA (Consortium of Investigators of Modifiers of *BRCA1/2*) [36] and OTTA (Ovarian Tumour Tissue Analysis) [37] consortia, the AOCS (Australian Ovarian Cancer Study) study [38] and collaborators of the ENIGMA consortium [34]. Data were collected using a predefined variable template, requesting information on the gene affected, classification of the detected variant, tumour invasion, histology, stage (FIGO), grade, variant nomenclature, ethnicity, age at ovarian cancer diagnosis and age at breast cancer diagnosis (if any). To collect relevant data from published studies, a literature search was conducted within the PubMed database searching for keywords such as ‘ovarian cancer’ and/or ‘ovarian cancer histology’ in combination with ‘*BRCA1* and/or *BRCA2* frequency’ or ‘predisposition’ (Supplementary Table S1). A total of 20 published studies meeting the study’s selection criteria, comprising 7733 ovarian cancer cases subjected to germline *BRCA1* and *BRCA2* genetic testing, were used.

### Exclusion criteria

Of the data collected, sites with a proportion of HGSCs over 90% and/or studies where the selection was applied for *BRCA1* and *BRCA2* genetic testing based on HGSC or non-mucinous histology were excluded from the dataset to account for potential selection bias [11, 17, 38–41]. Sources with a high proportion of unknown histology (≥50%) were also excluded. Overlaps between consortia or study groups (CIMBA, ENIGMA) and published studies were removed. Finally, tumour data of unknown/unclear, inconsistent, adenocarcinoma histology (representing carcinomas that cannot be allocated with certainty within major categories) and serous of undefined tumour grade information, were removed. Data reported as ‘other’ were comprehensively reviewed when such information was available and reclassified into appropriate categories or excluded. Additionally, individuals with somatic pathogenic variants (if this information was provided) or reported VUS in the non-carrier group, were removed. The final dataset consisted of 10,373 cases (Supplementary Table S2).

### Statistical analyses

Statistical analyses performed in this study are summarised in Fig. 1. As part of the main analysis, ovarian tumour histology was assessed as a predictor of germline *BRCA1* or *BRCA2* pathogenic variant status by defining likelihood ratio (LR) estimates. Data were grouped for *BRCA1* carriers, *BRCA2* carriers and non-carriers (*BRCA*_*0*_), and histology prevalence was determined for each group. LRs were calculated for each histological subtype by comparing their frequency between *BRCA1* or *BRCA2* pathogenic variant carriers and non-carriers:

$$LR = \frac{{p_i}}{{p_0}}$$

where $$p_i = \frac{{BRCA_i}}{{{\sum} {BRCA_i} }}$$ and $$p_0 = \frac{{BRCA_o}}{{{\sum} {BRCA_0} }}$$

*BRCAi* = 1, 2 denotes the number of *BRCA1* or *BRCA2* pathogenic variant carriers, respectively, for a given histological subtype, and *BRCA*_*0*_ denotes the number of non-carriers for the same histological subtype.

The variance of ln(LR) was calculated following Koopman et al. [42]:

$$Var\left( {\ln \left( {LR} \right)} \right) = \frac{{1 - p_i}}{{p_i \times {\sum} {BRCA_i} }} + \frac{{1 - p_0}}{{p_o \times {\sum} {BRCA_o} }}$$

Assuming a normal distribution for ln(LR), a 95% Confidence Interval (CI) was determined to assess the significance of the LR estimates obtained by:

$${{{{{{{\mathrm{CI}}}}}}}} = {{{{{{{\mathrm{Exp}}}}}}}}\left[ {{{{{{{{\mathrm{ln}}}}}}}}\left( {{{{{{{{\mathrm{LR}}}}}}}}} \right) \pm 1.96\sqrt {\left( {{{{{{{{\mathrm{Var}}}}}}}}\left( {{{{{{{{\mathrm{ln}}}}}}}}\left( {{{{{{{{\mathrm{LR}}}}}}}}} \right)} \right)} \right)} } \right]$$

Significant LR estimates, i.e., not spanning 1, suggested nominal significance, and potential for use as evidence following the ACMG/AMP system or the Multifactorial Likelihood model.

Using the same method, additional analyses were conducted, to assess differences in histological associations between clinically- or research-tested data and literature-derived data, as well as compare histological associations between Asian and European-origin ancestries. Other races and ancestries, including Hispanic (*N* = 302) and African (*N* = 14), did not provide informative predictions due to the low number of tumour data points available. Furthermore, histological associations were refined by other tumour and/or patient characteristics. First, tumour grade characteristics were refined as appropriate for each histotype. Mucinous, endometrioid, and ‘other’ histological subtypes were categorised as grade 1 (well-differentiated), grade 2 (moderately differentiated) and grade 3 (undifferentiated or poorly differentiated). Serous tumours, i.e., HGSC and LGSC were already separated according to a two-tier system. Clear cell was not refined by grade, since they are, by definition, high-grade [3, 43]. Histological subtype data were also combined with known information on tumour invasion (invasive or borderline). Due to the small number of borderline cases collected, borderline tumours were assessed separately as a single category without considering histology. Finally, age at ovarian cancer diagnosis (before and at/after the age of 50 years) was assessed in combination with histological subtype information, where tumours of unknown age at diagnosis were removed.

### ACMG/AMP LR evidence strength alignment

To determine the strength of the associations derived, LR values were aligned to the evidence values of the Bayesian framework of the ACMG/AMP system [32]. The strengths favouring variant pathogenicity included: very strong pathogenic, LR ≥ 350; strong pathogenic, 18.70 ≤ LR <350; moderate pathogenic, 4.33 ≤ LR <18.70; and supporting pathogenic, 2.08 ≤ LR <4.33. Evidence against variant pathogenicity were inferred using the inverse of the ranges proposed for the pathogenic strength evidence: very strong benign, LR < 0.00285; strong benign, 0.00285 ≤ LR < 0.053; moderate benign, 0.053 ≤LR <0.23; and supporting benign, 0.23 ≤LR < 0.48. We defined informative evidence as associations with statistically significant CI (i.e., not including 1). Categories having LR values within the range of 0.48 ≤ LR < 2.08 were referred as non-informative.

## Results

### Clinicopathological characteristics

The assembled dataset consisted of 10,373 ovarian cancer cases, including 2044 germline *BRCA1* carriers, 761 germline *BRCA2* carriers and 7568 non-carriers (based on germline testing) (Supplementary Table S2). Patient clinicopathological characteristics are shown in Table 1. The most frequent histotype was HGSC (70.9%), followed by endometrioid (9.7%), clear cell (6.3%), LGSC (4.9%), ‘other’ (4.7%), and mucinous (3.5%) histotypes. In Supplementary Table S3, histological subtypes are separated by tumour stage (FIGO), grade and age range at ovarian cancer diagnosis. Patient age at ovarian cancer diagnosis ranged from 18 to 92 years.

**Table 1:** Clinicopathological characteristics of the ovarian cancer case data collected.

Clinicopathological characteristics	*BRCA1* carriers, N (%)	*BRCA2* carriers, N (%)	Non-carriers, N (%)	Total N (%)
Total data	2044	761	7568	10,373
Age in years at OC diagnosis				
<30	6 (0.4)	5 (0.8)	105 (3.4)	119 (2.2)
30–39	170 (9.9)	11 (1.8)	245 (7.9)	426 (7.9)
40–49	670 (39.1)	102 (17.0)	563 (18.2)	1335 (24.7)
50–59	559 (32.6)	215 (35.8)	964 (31.2)	1738 (32.1)
60–69	251 (14.6)	199 (33.2)	833 (27.0)	1283 (23.7)
>70	58 (3.4)	68 (11.3)	379 (12.3)	505 (9.3)
N/A	330	161	4479	4970
Tumour grade				
Grade 1	55 (3.5)	29 (5.4)	401 (14.7)	485 (10.1)
Grade 2	281 (18.0)	91 (16.9)	389 (14.3)	762 (15.8)
Grade 3	1223 (78.4)	418 (77.7)	1934 (71.0)	3575 (74.1)
N/A	485	223	4846	5554
Tumour histology				
HGSC	1578 (77.2)	597 (78.4)	5183 (68.5)	7358 (70.9)
LGSC	58 (2.8)	23 (3.0)	429 (5.7)	510 (4.9)
Mucinous	21 (1.0)	14 (1.8)	325 (4.3)	361 (3.5)
Endometrioid	226 (11.1)	65 (8.5)	713 (9.4)	1004 (9.7)
Clear cell	38 (1.9)	15 (2.0)	605 (8.0)	658 (6.3)
‘Other’	123 (6.0)	47 (6.2)	313 (4.1)	485 (4.7)
Tumour Invasion				
Invasive	1468 (99.5)	530 (99.1)	2755 (94.8)	4754 (96.6)
Borderline	7 (0.5)	5 (0.9)	152 (5.2)	166 (3.4)
N/A	569	226	4661	5456

N number of data points, *OC* ovarian cancer, *HGSC* high-grade serous carcinomas, *LGSC* low-grade serous carcinomas, *N/A* not available.

The above data are based on 10,373 cases, including 2044 *BRCA1* carriers, 761 *BRCA2* carriers and 7568 non-carriers. In brackets, the frequency of the clinicopathological characteristics in all each group with known information is provided. The ‘other’ category denominates rare forms of ovarian cancer not belonging to any of the other subtypes, including tumours defined as: ‘other’ by data sources not specifying tumour histology; mixed-epithelial; carcinosarcomas; transitional cell (Brenner tumours); undifferentiated or poorly differentiated; squamous cell.

### Tumour histology association analysis

Ovarian cancer histological subtypes were assessed for their potential utility in future prediction of germline *BRCA1* or *BRCA2* variant pathogenicity. Detailed LR estimates derived are provided in Table 2. Under the ACMG/AMP system, no histological subtype provided informative evidence in favour of *BRCA1* and *BRCA2* variant pathogenicity. Evidence against *BRCA1* pathogenicity was estimated for the mucinous (LR: 0.24 (95% CI: 0.15–0.37), supporting evidence) and clear cell (LR: 0.23 (95% CI: 0.17–0.32), supporting evidence) histotypes. Similarly, evidence against *BRCA2* variant pathogenicity was derived for the mucinous (LR: 0.43 (95% CI: 0.25–0.73), supporting evidence) and clear cell histological subtypes (LR: 0.25 (95% CI: 0.15–0.41), supporting evidence). Histotypes failing to provide informative ACMG/AMP evidence, provided statistically significant LR estimates, suggestive of suitability to be included in Multifactorial Likelihood modelling (where there are no limitations set for individual LRs included). Specifically, LR estimates in favour of pathogenicity were identified for the HGSC and ‘other’ histotypes for *BRCA1* and *BRCA2* and the endometrioid histotype for *BRCA1*. Evidence against pathogenicity was also identified for LGSC for *BRCA1* and *BRCA2*.

**Table 2:** Likelihood ratio analysis for the evaluation of ovarian cancer histological subtypes in association with *BRCA1* and *BRCA2* pathogenic variant status.

	*BRCA1* carriers			*BRCA2* carriers			Non-carriers	Total
Histological subtypes	N (%)	LR (95% CI)	ACMG/AMP strength	N (%)	LR (95% CI)	ACMG/AMP strength	N (%)	N (%)
HGSC	1578 (77.2)	1.13 (1.10–1.16)	Non-informative	597 (78.4)	1.15 (1.10–1.19)	Non-informative	5183 (68.5)	7358 (70.9)
LGSC	58 (2.8)	0.50 (0.38–0.66)	Non-informative	23 (3.0)	0.53 (0.35–0.81)	Non-informative	429 (5.7)	510 (4.9)
Mucinous	21 (1.0)	**0.24 (0.15–0.37)**	**Supporting Benign**	14 (1.8)	**0.43 (0.25–0.73)**	**Supporting Benign**	325 (4.3)	360 (3.5)
Endometrioid	226 (11.1)	1.17 (1.02–1.35)	Non-informative	65 (8.5)	0.91 (0.71–1.16)	Non-informative	713 (9.4)	1004 (9.7)
Clear cell	38 (1.9)	**0.23 (0.17–0.32)**	**Supporting Benign**	15 (2.0)	**0.25 (0.15–0.41)**	**Supporting Benign**	605 (8.0)	658 (6.3)
‘Other’	123 (6.0)	1.45 (1.19–1.78)	Non-informative	47 (6.2)	1.49 (1.11–2.01)	Non-informative	313 (4.1)	483 (4.7)
	2044			761			7568	10,373

N number of data points, *LR* likelihood ratio, *CI* confidence interval, *ACMG/AMP* American College of Medical Genetics/Association for Molecular Pathology, *HGSC* high-grade serous carcinomas, *LGSC* low-grade serous carcinomas

The analysis was based on 10,373 cases, including 2044 *BRCA1* carriers, 761 *BRCA2* carriers and 7568 non-carriers. In brackets, the histotype frequency for each group is provided. The ‘other’ category denominates rare forms of ovarian cancer not belonging to any of the other subtypes, including tumours defined as: ‘other’ by data sources not specifying tumour histology; mixed-epithelial; carcinosarcomas; transitional cell (Brenner tumours); undifferentiated or poorly differentiated; squamous cell. LR > 1: Histotype association with pathogenic variant, Pathogenic evidence; LR < 1: Prediction of non-carrier for pathogenic variant, Benign evidence. Evidence strength was measured based on Bayesian modelling of ACMG/AMP rules (see 'Materials and methods'); Supporting Benign (LR ≥ 0.23–0.48), Moderate Benign (LR ≥ 0.053–0.23), Supporting Pathogenic (LR ≥ 2.08–4.30), non-informative (0.48 ≤ LR ≤ 2.08). LR estimates reaching informative ACMG/AMP strengths at a statistically significant CI (i.e., not spanning 1), are highlighted in bold.

Histological subtype associations also were compared between clinically derived and literature-derived data, to determine any major differences (Supplementary Table S4). Not all subtypes provided sufficient occurrences in this stratified dataset for informative comparisons. Overall, differences in ACMG/AMP code strength were observed for LGSC for *BRCA1* and *BRCA2* and mucinous for *BRCA2*, but these estimates, for literature-derived data in particular, were based on a small number of cases in each category, and confidence intervals for LR estimates overlapped.

Evaluation of the associations also were assessed by ancestry, by comparing the results of Asian- and European-ancestry data separately (Supplementary Table S5). Considering that the Asian-ancestry dataset was much smaller, no meaningful differences were observed in the direction of effect for LR estimates between the two sets, with the exception of the mucinous histotype which was not reported in Asian-origin *BRCA1* carriers. Results of the European-origin data alone, agreed with the LR estimates derived in the main analysis, with the addition of LGSC providing evidence against *BRCA1* variant pathogenicity (LR: 0.45 (95% CI: 0.33–0.60), supporting evidence).

### Assessing combined ovarian tumour characteristics

Histology associations with *BRCA1* and *BRCA2* variant pathogenicity were further refined by performing the LR analyses in combination with other ovarian tumour characteristics. First, histological categories were refined by tumour grade (Supplementary Table S6). Refinement resulted in a small amount of data within some categories, for which estimates should be used with caution. Endometrioid tumours of well-differentiated grade provided evidence against VUS pathogenicity for *BRCA1* (LR: 0.12 (95% CI: 0.04–0.31), moderate evidence) and *BRCA2* (LR: 0.31 (95% CI: 0.12–0.84), supporting evidence). In contrast, poorly differentiated endometrioid tumours were associated with evidence in favour of *BRCA1* variant pathogenicity (LR: 2.98 (95% CI: 2.28–3.89), supporting evidence) and *BRCA2* variant pathogenicity (LR: 2.09 (95% CI: 1.37–3.21), supporting evidence). Finally, the ‘other’ category provided informative evidence towards variant pathogenicity for both *BRCA1* (LR: 3.62 (95% CI: 2.61–5.03), supporting evidence) and *BRCA2* (LR: 3.46 (95% CI: 2.20–5.43), supporting evidence), if tumours were undifferentiated or poorly differentiated. Histology was integrated with information on tumour invasion (invasive or borderline) (Supplementary Table S7). Here, borderline tumours, irrespective of histology, were associated with moderate ACMG/AMP evidence against variant pathogenicity for *BRCA1* (LR: 0.09 (95% CI: 0.04–0.20)) and *BRCA2* (LR: 0.19 (95% CI: 0.08–0.46)). When invasive histological subtypes were assessed, similar LR estimates were obtained as in the main analysis. In addition, evidence against pathogenicity increased from supporting to moderate strength for invasive mucinous and clear cell tumours for *BRCA1*, and for invasive clear cell tumours for *BRCA2*. Also, invasive LGSC was associated with evidence against *BRCA1* pathogenicity at supporting strength (LR: 0.44 (95% CI: 0.31–0.62)). Histology-derived LRs also were estimated when categorised by age at ovarian cancer diagnosis (Supplementary Table S8). LGSC presentation before age 50 years provided evidence against pathogenicity for *BRCA1* (LR: 0.18 (95% CI: 0.11–0.30), moderate strength). Mucinous tumour presentation provided somewhat greater evidence for the association against *BRCA1* variant pathogenicity when the diagnosis was before age 50 years (LR: 0.11 (95% CI: 0.06–0.19), moderate evidence), compared to diagnosis at/after the age of 50 (LR: 0.21 (95% CI: 0.10–0.44), moderate evidence). Similarly, clear cell tumours provided somewhat greater evidence against *BRCA1* variant pathogenicity before the age of 50 (LR: 0.16 (95% CI: 0.08–0.31), moderate evidence) versus over that age (LR: 0.37 (95% CI: 0.23–0.59), supporting evidence). For *BRCA2*, evidence against pathogenicity was reached for LGSC in individuals diagnosed before the age of 50 years (LR: 0.43 (95% CI: 0.19–0.96), supporting evidence). Mucinous tumours provided supporting evidence against *BRCA2* variant pathogenicity in individuals diagnosed both before the age of 50 (LR: 0.36 (95% CI: 0.16–0.80), supporting evidence) and after the age of 50 (LR: 0.42 (95% CI: 0.21–0.87), supporting evidence). Likewise, clear cell tumour phenotype provided supporting evidence against pathogenicity at/after the age of 50 (LR: 0.30 (95% CI: 0.15–0.58)).

Based on the patient’s available information and based on which characteristic(s) were clinically informative in different sub-analyses, we propose that LR estimates and corresponding ACMG/AMP evidence are applied only for the characteristics presented in Table 3.

**Table 3:** Proposed application of ovarian cancer pathology characteristics for the interpretation of germline *BRCA1* and *BRCA2* variants, using the ACMG/AMP system*.

Tumour pathology		*BRCA1* evidence		*BRCA2* evidence		
Histology	Additional characteristics (If applicable)	ACMG/AMP strength	LR (95% CI)	ACMG/AMP strength	LR (95% CI)	Source
LGSC	Age at diagnosis <50 y	Moderate benign	0.18 (0.11–0.30)	Supporting Benign	0.43 (0.19–0.96)	Supplementary Table S8
	Invasive, age unspecified	Supporting benign	0.44 (0.31–0.62)	–	–	Supplementary Table S7
Mucinous	–	Supporting benign	0.24 (0.15–0.37)	Supporting Benign	0.43 (0.25–0.73)	Table 2
Endometrioid	Grade 1	Moderate benign	0.12 (0.04–0.31)	Supporting Benign	0.31 (0.12–0.84)	Supplementary Table S6
	Grade 3	Supporting pathogenic	2.98 (2.28–3.89)	Supporting Pathogenic	2.09 (1.37–3.21)	Supplementary Table S6
Clear cell	–	Supporting benign	0.23 (0.17–0.32)	Supporting Benign	0.25 (0.15–0.41)	Table 2
Borderline	–	Moderate benign	0.09 (0.04–0.20)	Moderate Benign	0.19 (0.08–0.46)	Supplementary Table S7

*LR* likelihood ratio, *CI* confidence interval, *ACMG/AMP* American College of Medical Genetics/Association for Molecular Pathology, *LGSC* low-grade serous carcinomas, *y* years.

Evidence strength was measured based on Bayesian modelling of ACMG/AMP rules (see 'Materials and methods').

*Only associations reaching informative ACMG/AMP strengths at a statistically significant CI (i.e., not spanning 1), of which the characteristics were clinically informative, are shown. All other tumour histotypes (irrespective of additional characteristics) are considered uninformative for variant interpretation.

## Discussion

In this multicentre study, we evaluated the association of ovarian tumour histology with germline *BRCA1* and *BRCA2* pathogenic variant status. We aimed to standardise the application of this evidence in clinical variant curation using the ACMG/AMP classification system, to inform future VUS interpretation in *BRCA1* and *BRCA2*.

Following the alignment to the ACMG/AMP evidence strengths, no associations were derived in favour of *BRCA1* and *BRCA2* variant pathogenicity for the ovarian cancer histotypes analysed (Table 2). Nominal associations in favour of pathogenicity for the HGSC histotype may be suitable for inclusion in Multifactorial Likelihood modelling. This weak evidence reflects the high percentage of HGSC in ovarian cancer, irrespective of the presence of germline *BRCA1* and *BRCA2* pathogenic variants [1]. Evidence against pathogenicity at supporting strength was derived for *BRCA1* and *BRCA2* for the mucinous and clear cell histologies. Furthermore, evidence against pathogenicity for some categories, even though they may not reach ACMG/AMP strengths, could be used for inclusion in Multifactorial Likelihood modelling.

Sensitivity analyses exploring the heterogeneity within the dataset indicated that the inclusion of clinically- or research-collected data and data from published sources was unlikely to have caused any major confounding within the dataset. No significant differences were observed when comparing associations between data of different ancestries. However, the small data sizes of non-European ancestry data did not allow for reliable predictions and may not be generalisable.

In addition, we performed a series of refined histological subgroup analyses with the aim of incorporating additional information in the ovarian cancer pathology component of variant interpretation. Briefly, clinically informative predictions were derived for the endometrioid histology when separated by grade. Well-differentiated endometrioid tumour subtype was associated with evidence against *BRCA1* and *BRCA2* variant pathogenicity. Undifferentiated or poorly differentiated endometrioid tumour subtypes were associated with evidence in favour of variant pathogenicity, which likely reflects a proportion of misclassified HGSCs [44]. Although we observed an association in favour of pathogenicity of the ‘other’ subtype category when combined with the grade, we do not recommend use in clinical practice due to the high possibility of data misclassification within this category (HGSC or undefined/unknown histology often miscalled as ‘other’ (12.7% of ‘other’ category are poorly differentiated/undifferentiated which are often miscalled as HGSC cases). The association of this category with *BRCA1* and *BRCA2* variant pathogenicity should be evaluated in future studies. Refined analyses also suggested that borderline phenotype (irrespective of histology) was associated with evidence against *BRCA1* and *BRCA2* variant pathogenicity at moderate strength. Therefore, despite the rare occurrence of mucinous or borderline characteristics in carriers of pathogenic *BRCA1* and *BRCA2* variants, the phenotypes have a clinical value in informing VUS interpretation. Since the majority of data for borderline cases were of mucinous histology, the identification of such evidence is consistent with earlier observations for the histotype. Refinement by invasive designation also informed predictions against variant pathogenicity. Lastly, when age at diagnosis was considered, clinically informative predictions were identified for LGSC histotype diagnosis before the age of 50 years, providing evidence against *BRCA1* and *BRCA2* variant pathogenicity.

Based on the above results, we propose a strategy for the use of ovarian tumour histology in the assessment of germline *BRCA1* and *BRCA2* VUS interpretation under the strict rules of the ACMG/AMP system, based on patient characteristics and available information (Table 3). This information may be used in combination with other evidence to inform variant classification, and subsequent patient and family management. The identified LR estimates (Table 2) may also be used directly within the Multifactorial Likelihood model. Note that, optimally, ascertainment criterion for genetic testing of the carrier should be considered when applying LRs (e.g., testing for only HGSC). Our study also provides a demonstration on the use of statistical likelihood ratio modelling for the evaluation of associations of tumour characteristics and variant pathogenicity, with applicability to inform variant interpretation in other tumour types and genes.

We would like to acknowledge the following caveats. Although we tried to minimise the effect of potential selection for *BRCA1* and *BRCA2* clinical testing based on histological phenotype, we cannot discount the possibility of selection in individual sites. Studies applying selection for *BRCA1* and *BRCA2* genetic testing based on non-mucinous histology were excluded from the main analysis [11, 17, 38–41]. A separate analysis including these data (an additional 159 *BRCA1* carriers, 101 *BRCA2* carriers and 982 non-carriers) did not materially change our predictions; findings suggest that the LRs from the main analysis will be applicable in the context of non-mucinous testing, although it should be noted that the additional data points per histological category were relatively few except for HGSC (data not shown). Finally, although the data collection requirements specified the inclusion of pathogenic or likely pathogenic variant carriers, due to the absence of detailed variant information for data evaluation, and due to changes in classification practices over time, we cannot exclude the possibility that some variants might be misclassified. Furthermore, due to the wide confidence intervals for some of the subtype-specific results, a more conservative approach might be required before the use of these evidence categories in the clinical classification of variants. Overall, it is likely that the practical application of LRs for future variant interpretation will provide additional insight into their correlation with existing clinical and functional evidence types already commonly used in *BRCA1* and *BRCA2* variant interpretation.

## Conclusion

In this study, ovarian cancer histological subtypes were evaluated as predictors of *BRCA1* and *BRCA2* pathogenic variant status. We also provided refined LR estimates for the association of ovarian cancer histology in combination with other tumour and patient characteristics. Overall, we provide evidence for the incorporation of the derived LR estimates in variant classification to improve the interpretation of VUS identified in the *BRCA1* and *BRCA2*, and thereby inform carrier clinical management.

## Supplementary information

Supplementary Material

## Data Availability

All data generated in this study can be found in the Supplementary Material file.
