# Supplementary material for: Ovarian cancer pathology characteristics as predictors of variant pathogenicity in BRCA1 and BRCA2
Source: Br J Cancer. 2023 Apr 19;128(12):2283–94. doi: 10.1038/s41416-023-02263-5 (PMC10241792; doi:10.1038/s41416-023-02263-5)
Supplement: Supplementary file 1 — Supplementary Material [file 41416_2023_2263_MOESM1_ESM.docx]

**OVARIAN CANCER PATHOLOGY CHARACTERISTICS AS PREDICTORS OF VARIANT PATHOGENICITY IN *BRCA1* AND *BRCA2***

Supplementary Material guide

**Supplementary Table S1.** **Literature studies reviewed for current study and study selection process**

Description of published studies found by literature search. File shows the studies included in the final dataset and the corresponding data used as well as the studies excluded and reason for exclusion.

**Supplementary Table S2.** **Ovarian cancer data collected from relevant sources, according to histology subtype and carrier status**

Final dataset assembled after data exclusion from all sources. The number of samples collected per histological subtype in *BRCA1* carriers, *BRCA2* carriers, non-carriers and total samples of the dataset are given for each study/site.

**Supplementary Table S3.** **Characterisation of histological subtypes according to tumour stage, grade and age at ovarian cancer diagnosis**

Histological subtypes separated by tumour stage (FIGO), grade and age range at ovarian cancer diagnosis.

**Supplementary Table S4.** **Clinical-tested or research-tested data and literature data likelihood ratio association analyses of ovarian tumour histotypes**

Histological subtype associations with *BRCA1* and *BRCA2* variant pathogenicity by Likelihood ratio analysis, separated for clinically/research-derived data and literature-derived data.

**Supplementary Table S5.** **European- and Asian-origin ancestry likelihood ratio association analyses of ovarian tumour histotypes**

Histological subtype associations with *BRCA1* and *BRCA2* variant pathogenicity by Likelihood ratio analysis, separated for European-origin data and Asian-origin data.

**Supplementary Table S6.** **Likelihood ratio association analysis based on ovarian tumour histology and tumour grade**

Histological subtype associations with *BRCA1* and *BRCA2* variant pathogenicity by Likelihood ratio analysis refined by tumour grade (grade 1, 2 or 3).

**Supplementary Table S7.** **Likelihood ratio association analysis based on ovarian tumour invasion and tumour histology**

Histological subtype associations with *BRCA1* and *BRCA2* variant pathogenicity by Likelihood ratio analysis refined by tumour invasion (borderline tumours separated from invasive tumours).

**Supplementary Table S8.** **Likelihood ratio association analysis based on ovarian tumour histology and age at diagnosis**

Histological subtype associations with *BRCA1* and *BRCA2* variant pathogenicity by Likelihood ratio analysis refined by patient age at diagnosis (before or at/after the age of 50).

**Supplementary Table S1**

**Literature studies reviewed for current study and study selection process**

| Literature study | PMID | Title | Inclusion in study  (yes/no) | Reason for exclusion  (If applicable) | Type of data used   (Regarding *BRCA1/2* pathogenic variant status) | Additional Information  (e.g., selection criteria, issue in dataset) |
| --- | --- | --- | --- | --- | --- | --- |
| Hirasawa  et al. 2017 | PMID: 29348823 | Prevalence of pathogenic germline variants detected by multigene sequencing in unselected Japanese patients with ovarian cancer | **yes** | - | All data | - |
| Seo  et al. 2019 | PMID: 31777737 | Prevalence and oncologic outcomes of *BRCA1/2* mutation and variant of unknown significance in epithelial ovarian carcinoma patients in Korea | **yes** | - | All data | - |
| Li  et al. 2019 | PMID: 31472684 | Germline and somatic mutations of multi-gene panel in Chinese patients with epithelial ovarian cancer: a prospective cohort study | **yes** | - | All data | - |
| Norquist  et al. 2016 | [PMID: 26720728](https://pubmed.ncbi.nlm.nih.gov/26720728) | Inherited Mutations in Women with Ovarian Carcinoma | **yes** | - | All data | - |
| Sakamoto  et al. 2015 | PMID: 26439132 | *BRCA1* and *BRCA2* mutations in Japanese patients with ovarian, fallopian tube, and primary peritoneal cancer | **yes** | - | All data | - |
| Sugino  et al. 2019 | [PMID: 31780705](https://pubmed.ncbi.nlm.nih.gov/31780705) | Germline and somatic mutations of homologous recombination-associated genes in Japanese ovarian cancer patients | **yes** | - | All data | - |
| Kowalik  et al. 2019 | PMID: 31556562 | Somatic mutations in *BRCA1*&2 in 201 unselected ovarian carcinoma samples - single institution study | **yes** | - | *BRCA1* or *BRCA2* carriers | - |
| Hilton  et al. 2002 | PMID: 12237285 | Inactivation of *BRCA1* and *BRCA2* in ovarian cancer | **yes** | - | *BRCA2* carriers | - |
| Ataseven  et al. 2020 | PMID: 32905297 | Prevalence of *BRCA1* and *BRCA2* Mutations in Patients with Primary Ovarian Cancer - Does the German Checklist for Detecting the Risk of Hereditary Breast and Ovarian Cancer Adequately Depict the Need for Consultation? | **yes** | - | Non-carriers | Joined *BRCA1/2* data |
| Bu  et al. 2019 | PMID: 31411802 | *BRCA* mutation frequency and clinical features of ovarian cancer patients: A report from a Chinese study group | **yes** | - | Non-carriers | Joined *BRCA1/2* data |
| Kim  et al. 2020 | PMID: 32718143 | Germline and Somatic *BRCA1/2* Gene Mutational Status and Clinical Outcomes in Epithelial Peritoneal, Ovarian, and Fallopian Tube Cancer: Over a Decade of Experience in a Single Institution in Korea | **yes** | - | Non-carriers | Joined *BRCA1/2* data |
| Wu  et al. 2017 | PMID: 28692638 | The First Nationwide Multicenter Prevalence Study of Germline *BRCA1* and *BRCA2* Mutations in Chinese Ovarian Cancer Patients | **yes** | - | Non-carriers | Joined *BRCA1/2* data |
| Sarantaus  et al. 2001 | PMID: 11251181 | *BRCA1* and *BRCA2* mutations among Finnish ovarian carcinoma families | **yes** | - | All data | Non-borderline phenotype selection |
| Vos  et al. 2020 | PMID: 31076742 | Universal Tumor DNA *BRCA1/2* Testing of Ovarian Cancer: Prescreening PARPi Treatment and Genetic Predisposition | **yes** | - | All data | Non-borderline phenotype selection |
| Brozek  et al. 2008 | PMID: 17997147 | High frequency of *BRCA1/2* germline mutations in consecutive ovarian cancer patients in Poland | **yes** | - | *BRCA1* or *BRCA2* carriers | Non-borderline phenotype selection |
| Johansson  et al. 1998 | PMID: 9469321 | Survival of *BRCA1* breast and ovarian cancer patients: a population-based study from southern Sweden | **yes** | - | *BRCA1* carriers | Non-borderline phenotype selection, *BRCA1* data only |
| Soegaard  et al. 2008 | PMID: 18559594 | *BRCA1* and *BRCA2* mutation prevalence and clinical characteristics of a population-based series of ovarian cancer cases from Denmark | **yes** | - | *BRCA1* or *BRCA2* carriers | Non-borderline phenotype selection, no differentiation between HGSC & LGSC in non-carriers (non-carrier data not used) |
| Khoo  et al. 2000 | PMID: 10874312 | Mutational analysis of *BRCA1* and *BRCA2* genes in Chinese ovarian cancer identifies 6 novel germline mutations | **yes** | - | *BRCA1* or *BRCA2* carriers | Recurrent mutations (#5) in the Chinese population |
| Bernards  et al. 2016 | [PMID: 26718727](https://pubmed.ncbi.nlm.nih.gov/26718727) | Genetic characterization of early onset ovarian carcinoma | **yes** | - | All data | Selection based on age at diagnosis <40 years of age |
| Enomoto  et al. 2019 | PMID: 31263023 | The first Japanese nationwide multicenter study of *BRCA* mutation testing in ovarian cancer: CHARacterizing the cross-sectionaL approach to Ovarian cancer geneTic TEsting of *BRCA* (CHARLOTTE) | **yes** | - | All data | Undefined 'other' category |
| Adams  et al. 2011 | PMID: 21945552 | A high response rate to liposomal doxorubicin is seen among women with *BRCA* mutations treated for recurrent epithelial ovarian cancer | no | Undefined whether somatic or germline | - | - |
| Gotlieb  et al. 2005 | PMID: 15893369 | Demographic and genetic characteristics of patients with borderline ovarian tumors as compared to early-stage invasive ovarian cancer | no | Serous vs non-Serous, No differentiation between HGSC & LGSC | - | - |
| Chetrit  et al. 2008 | PMID: 18165636 | Effect of *BRCA1/2* mutations on long-term survival of patients with invasive ovarian cancer: the national Israeli study of ovarian cancer | no | Serous vs non-Serous | - | - |
| Shaw  et al. 2002 | PMID: 12352190 | Histopathologic features of genetically determined ovarian cancer | no | Serous vs non-Serous | - | - |
| Belanger  et al. 2015 | PMID: 25884701 | A targeted analysis identifies a high frequency of *BRCA1* and *BRCA2* mutation carriers in women with ovarian cancer from a founder population | no | Selection for HGSC, Founder mutations only | - | - |
| Lhotova  et al. 2020 | PMID: 32295079 | Multigene Panel Germline Testing of 1333 Czech Patients with Ovarian Cancer | no | Received clinical data, so included in the clinical dataset assembled | - | - |
| Walsh  et al. 2011 | PMID: 22006311 | Mutations in 12 genes for inherited ovarian, fallopian tube, and peritoneal carcinoma identified by massively parallel sequencing | no | Potential overlap with Norquist et al. 2016 | - | - |
| Bolton  et al. 2012 | [PMID: 22274685](https://pubmed.ncbi.nlm.nih.gov/22274685) | Association between *BRCA1* and *BRCA2* mutations and survival in women with invasive epithelial ovarian cancer | no | Potential overlap with CIMBA, No differentiation between HGSC & LGSC | - | - |
| George  et al. 2016 | PMID: 27406733 | Implementing rapid, robust, cost-effective, patient-centred, routine genetic testing in ovarian cancer patients | no | Non-mucinous selection, potential overlap with CIMBA | - | - |
| Peixoto  et al. 2020 | [PMID: 32850417](https://pubmed.ncbi.nlm.nih.gov/32850417) | Tumor Testing for Somatic and Germline *BRCA1*/ *BRCA2* Variants in Ovarian Cancer Patients in the Context of Strong Founder Effects | no | Non-mucinous selection, potential overlap with CIMBA | - | - |
| Berchuck  et al. 1998 | PMID: 9796975 | Frequency of germline and somatic *BRCA1* mutations in ovarian cancer | no | Non-mucinous selection | - | - |
| Choi  et al. 2015 | PMID: 19499246 | *BRCA1* and *BRCA2* germline mutations in Korean ovarian cancer patients | no | Non-mucinous selection | - | - |
| Rudaitis  et al. 2014 | PMID: 25248112 | *BRCA1/2* mutation status is an independent factor of improved survival for advanced (stage III-IV) ovarian cancer | no | Non-mucinous histopathology selection, No differentiation between HGSC & LGSC | - | - |
| Hennessy  et al. 2010 | PMID: 20606085 | Somatic mutations in *BRCA1* and *BRCA2* could expand the number of patients that benefit from poly (ADP ribose) polymerase inhibitors in ovarian cancer | no | No differentiation between HGSC & LGSC, Serous vs non-Serous | - | - |
| Lertkhachonsuk et al. 2020 | PMID: 32856869 | Prevalence of Tissue *BRCA* Gene Mutation in Ovarian, Fallopian Tube, and Primary Peritoneal Cancers: A Multi-Institutional Study | no | Selection for HGSC, High Grade endometrioid or clear cell | - | - |
| Safra  et al. 2011 | PMID: 21835933 | *BRCA* mutation status and determinant of outcome in women with recurrent epithelial ovarian cancer treated with pegylated liposomal doxorubicin | no | No differentiation between HGSC & LGSC, Selection for HGSC & endometrioid | - | - |
| Lu  et al. 1999 | PMID: 9916952 | A population-based study of *BRCA1* and *BRCA2* mutations in Jewish women with epithelial ovarian cancer | no | No differentiation between HGSC & LGSC, Founder mutations only | - | - |
| Moslehi  et al. 2000 | PMID: 10739756 | *BRCA1* and *BRCA2* mutation analysis of 208 Ashkenazi Jewish women with ovarian cancer | no | No differentiation between HGSC & LGSC, Founder mutations only | - | - |
| Rafnar  et al. 2004 | PMID: 15571962 | *BRCA2*, but not *BRCA1*, mutations account for familial ovarian cancer in Iceland: a population-based study | no | No differentiation between HGSC & LGSC, Founder mutations only | - | - |
| Tonin  et al. 1999 | PMID: 11307153 | Founder *BRCA1* and *BRCA2* mutations in early-onset French Canadian breast cancer cases unselected for family history | no | No differentiation between HGSC & LGSC, Founder mutations only | - | - |
| Porto Cotrim et al. 2019 | PMID: 30606148 | Prevalence of *BRCA1* and *BRCA2* pathogenic and likely pathogenic variants in non-selected ovarian carcinoma patients in Brazil | no | No differentiation between HGSC & LGSC, Joined *BRCA1/2* data, Serous vs non-serous | - | - |
| Boyd  et al. 2000 | PMID: 10807385 | Clinicopathologic features of *BRCA*-linked and sporadic ovarian cancer | no | No differentiation between HGSC & LGSC, Joined *BRCA1/2* data | - | - |
| Deng  et al. 2019 | [PMID: 30972954](https://pubmed.ncbi.nlm.nih.gov/30972954) | Comprehensive analysis of serum tumor markers and *BRCA1/2* germline mutations in Chinese ovarian cancer patients | no | No differentiation between HGSC & LGSC, Joined *BRCA1/2* data | - | - |
| Ashour  et al. 2019 | [PMID: 31372034](https://pubmed.ncbi.nlm.nih.gov/31372034) | Frequency of germline mutations in *BRCA1* and *BRCA2* in ovarian cancer patients and their effect on treatment outcome | no | No differentiation between HGSC & LGSC, Joined *BRCA1/2* data | - | - |
| Chao  et al. 2016 | PMID: 27907908 | Prevalence and clinical significance of *BRCA1/2* germline and somatic mutations in Taiwanese patients with ovarian cancer | no | No differentiation between HGSC & LGSC, Joined *BRCA1/2* data | - | - |
| Rivera  et al. 2020 | PMID: 32895300 | Implementing NGS-based *BRCA* tumour tissue testing in FFPE ovarian carcinoma specimens: hints from a real-life experience within the framework of expert recommendations | no | No differentiation between HGSC & LGSC, Joined *BRCA1/2* data | - | - |
| Risch  et al. 2006 | PMID: 17148771 | Population *BRCA1* and *BRCA2* mutation frequencies and cancer penetrances: a kin-cohort study in Ontario, Canada | no | No differentiation between HGSC & LGSC, endometrioid and clear cell samples combined | - | - |
| Ang Li  et al. 2018 | PMID: 30078507 | *BRCA* germline mutations in an unselected nationwide cohort of Chinese patients with ovarian cancer and healthy controls | no | No differentiation between HGSC & LGSC | - | - |
| Dann  et al. 2012 | PMID: 22406760 | *BRCA1/2* mutations and expression: response to platinum chemotherapy in patients with advanced stage epithelial ovarian cancer | no | No differentiation between HGSC & LGSC | - | - |
| Evans  et al. 2008 | PMID: 18312450 | Probability of *BRCA1/2* mutation varies with ovarian histology: results from screening 442 ovarian cancer families | no | No differentiation between HGSC & LGSC | - | - |
| Fong  et al. 2010 | PMID: 20406929 | Poly (ADP)-ribose polymerase inhibition: frequent durable responses in *BRCA* carrier ovarian cancer correlating with platinum-free interval | no | No differentiation between HGSC & LGSC | - | - |
| Gallagher  et al. 2011 | PMID: 21084428 | Survival in epithelial ovarian cancer: a multivariate analysis incorporating *BRCA* mutation status and platinum sensitivity | no | No differentiation between HGSC & LGSC | - | - |
| Harter  et al. 2015 | PMID: 29506471 | A population-based analysis of germline *BRCA1* and *BRCA2* testing among ovarian cancer patients in an era of histotype-specific approaches to ovarian cancer prevention | no | No differentiation between HGSC & LGSC | - | - |
| Hoberg-Vetti et al. 2016 | PMID: 26350514 | *BRCA1/2* testing in newly diagnosed breast and ovarian cancer patients without prior genetic counselling: the DNA-BONus study | no | No differentiation between HGSC & LGSC | - | - |
| Kotsopoulos et al. 2015 | PMID: 25482078 | Factors influencing ovulation and the risk of ovarian cancer in *BRCA1* and *BRCA2* mutation carriers | no | No differentiation between HGSC & LGSC | - | - |
| Kringen  et al. 2005 | [PMID: 16229746](https://pubmed.ncbi.nlm.nih.gov/16229746) | TP53 mutations in ovarian carcinomas from sporadic cases and carriers of two distinct *BRCA1* founder mutations; relation to age at diagnosis and survival | no | No differentiation between HGSC & LGSC | - | - |
| Lacour  et al. 2011 | PMID: 21276604 | Improved survival in non-Ashkenazi Jewish ovarian cancer patients with *BRCA1* and *BRCA2* gene mutations | no | No differentiation between HGSC & LGSC | - | - |
| Lakhani  et al. 2004 | PMID: 15073127 | Pathology of ovarian cancers in *BRCA1* and *BRCA2* carriers | no | No differentiation between HGSC & LGSC | - | - |
| Lilyquist  et al. 2017 | PMID: 28888541 | Frequency of mutations in a large series of clinically ascertained ovarian cancer cases tested on multi-gene panels compared to reference controls | no | No differentiation between HGSC & LGSC | - | - |
| Liu  et al. 2012 | [PMID: 23057551](https://pubmed.ncbi.nlm.nih.gov/23057551) | Differing clinical impact of *BRCA1* and *BRCA2* mutations in serous ovarian cancer | no | No differentiation between HGSC & LGSC | - | - |
| Maistro  et al. 2016 | PMID: 27914478 | Germline mutations in *BRCA1* and *BRCA2* in epithelial ovarian cancer patients in Brazil | no | No differentiation between HGSC & LGSC | - | - |
| Majdak  et al. 2005 | PMID: 16047333 | Prognostic impact of *BRCA1* pathogenic and *BRCA1*/*BRCA2* unclassified variant mutations in patients with ovarian carcinoma | no | No differentiation between HGSC & LGSC | - | - |
| Malander  et al. 2004 | PMID: 14746861 | One in 10 ovarian cancer patients carry germ line *BRCA1* or *BRCA2* mutations: results of a prospective study in Southern Sweden | no | No differentiation between HGSC & LGSC | - | - |
| Pal  et al. 2005 | PMID: 16284991 | *BRCA1* and *BRCA2* mutations account for a large proportion of ovarian carcinoma cases | no | No differentiation between HGSC & LGSC | - | - |
| Pharoah  et al. 1999 | PMID: 10029077 | Survival in familial, *BRCA1*-associated, and *BRCA2*-associated epithelial ovarian cancer. United Kingdom Coordinating Committee for Cancer Research (UKCCCR) Familial Ovarian Cancer Study Group | no | No differentiation between HGSC & LGSC | - | - |
| Reitsma  et al. 2012 | PMID: 22274543 | Clinicopathologic characteristics and survival in *BRCA1*- and *BRCA2*-related adnexal cancer: are they different? | no | No differentiation between HGSC & LGSC | - | - |
| Risch  et al. 2001 | PMID: 17148771 | Population *BRCA1* and *BRCA2* mutation frequencies and cancer penetrances: a kin-cohort study in Ontario, Canada | no | No differentiation between HGSC & LGSC | - | - |
| Rubin  et al. 1996 | PMID: 8875917 | Clinical and pathological features of ovarian cancer in women with germ-line mutations of *BRCA1* | no | No differentiation between HGSC & LGSC | - | - |
| Sabatier  et al. 2016 | PMID: 26833043 | Ovarian cancer patients at high risk of *BRCA* mutation: the constitutional genetic characterization does not change prognosis | no | No differentiation between HGSC & LGSC | - | - |
| Safra  et al. 2014 | PMID: 24131973 | *BRCA* mutations and outcome in epithelial ovarian cancer (EOC): experience in ethnically diverse groups | no | No differentiation between HGSC & LGSC | - | - |
| Sekine  et al. 2001 | PMID: 11595708 | Mutational analysis of *BRCA1* and *BRCA2* and clinicopathologic analysis of ovarian cancer in 82 ovarian cancer families: two common founder mutations of *BRCA1* in Japanese population | no | No differentiation between HGSC & LGSC | - | - |
| Smith  et al. 2001 | PMID: 11733976 | *BRCA1* germline mutations and polymorphisms in a clinic-based series of ovarian cancer cases: a Gynecologic Oncology Group study | no | No differentiation between HGSC & LGSC | - | - |
| Song  et al. 2014 | PMID: 24728189 | The contribution of deleterious germline mutations in *BRCA1*, *BRCA2* and the mismatch repair genes to ovarian cancer in the population | no | No differentiation between HGSC & LGSC | - | - |
| Synowiec  et al. 2016 | [PMID: 26753012](https://pubmed.ncbi.nlm.nih.gov/26753012) | Clinical features and outcomes of germline mutation *BRCA1*-linked versus sporadic ovarian cancer patients | no | No differentiation between HGSC & LGSC | - | - |
| Unni  et al. 2016 | [PMID: 27004793](https://pubmed.ncbi.nlm.nih.gov/27004793) | *BRCA* testing, treatment patterns and survival in platinum-sensitive recurrent ovarian cancer - an observational cohort study | no | No differentiation between HGSC & LGSC | - | - |
| Vencken  et al. 2011 | PMID: 21228333 | Chemosensitivity and outcome of *BRCA1*- and *BRCA2*-associated ovarian cancer patients after first-line chemotherapy compared with sporadic ovarian cancer patients | no | No differentiation between HGSC & LGSC | - | - |
| Villareal-Garza  et al. 2015 | PMID: 25236687 | Significant clinical impact of recurrent *BRCA1* and *BRCA2* mutations in Mexico | no | No differentiation between HGSC & LGSC | - | - |
| Werness  et al. 2000 | PMID: 11112219 | Histopathology of familial ovarian tumors in women from families with and without germline *BRCA1* mutations | no | No differentiation between HGSC & LGSC | - | - |
| Werness  et al. 2004 | PMID: 14668547 | Histopathology, FIGO stage, and *BRCA* mutation status of ovarian cancers from the Gilda Radner Familial Ovarian Cancer Registry | no | No differentiation between HGSC & LGSC | - | - |
| Yamashita  et al. 1999 | PMID: 10634513 | *BRCA1* mutation testing for Japanese patients with ovarian cancer in breast cancer screening | no | No differentiation between HGSC & LGSC | - | - |
| Yazici  et al. 2002 | PMID: 12112655 | *BRCA1* and *BRCA2* mutations in Turkish familial and non-familial ovarian cancer patients: a high incidence of mutations in non-familial cases | no | No differentiation between HGSC & LGSC | - | - |
| Zhang  et al. 2011 | PMID: 21324516 | Frequencies of *BRCA1* and *BRCA2* mutations among 1,342 unselected patients with invasive ovarian cancer | no | No differentiation between HGSC & LGSC | - | - |
| Zweemer  et al. 2011 | PMID: 9465809 | Clinical and genetic evaluation of thirty ovarian cancer families | no | No differentiation between HGSC & LGSC | - | - |
| Jacobi  et al. 2017 | PMID: 17413421 | Prediction of *BRCA1/2* mutation status in patients with ovarian cancer from a hospital-based cohort | no | No differentiation between *BRCA1/2* and other BC genes, Joined *BRCA1/2* data | - | - |
| Shi  et al. 2017 | PMID: 28176296 | *BRCA1* and *BRCA2* mutations in ovarian cancer patients from China: ethnic-related mutations in *BRCA1* associated with an increased risk of ovarian cancer | no | No differentiation between *BRCA1/2* and other BC genes, Joined *BRCA1/2* data | - | - |
| Cunningham et al. 2014 | PMID: 24504028 | Clinical characteristics of ovarian cancer classified by *BRCA1*, *BRCA2*, and RAD51C status | no | No differentiation between *BRCA1/2* and other breast cancer genes | - | - |
| Flaum  et al. 2020 | PMID: 32651552 | Mainstreaming germline *BRCA1/2* testing in non-mucinous epithelial ovarian cancer in the North West of England | no | Joined *BRCA1/2* data, non-mucinous selection | - | - |
| Biglia  et al. 2015 | PMID: 27350785 | Ovarian cancer in *BRCA1* and *BRCA2* gene mutation carriers: analysis of prognostic factors and survival | no | Joined *BRCA1/2* data | - | - |
| Fumagalli  et al. 2022 | PMID: 35406410 | Tumor *BRCA* Testing in Epithelial Ovarian Cancers: Past and Future-Five-Years' Single-Institution Experience of 762 Consecutive Patients | no | Joined *BRCA1/2* data | - | - |
| Morgan  et al. 2019 | PMID: 30683677 | Prevalence of germline pathogenic *BRCA1/2* variants in sequential epithelial ovarian cancer cases | no | Joined *BRCA1/2* data | - | - |
| Fumagalli  et al. 2019 | PMID: 31653094 | Tumor *BRCA* Test for Patients with Epithelial Ovarian Cancer: The Role of Molecular Pathology in the Era of PARP Inhibitor Therapy | no | High-grade serous vs non-serous | - | - |
| Plaskocinska et al. 2016 | PMID: 27208206 | New paradigms for *BRCA1*/*BRCA2* testing in women with ovarian cancer: results of the Genetic Testing in Epithelial Ovarian Cancer (GTEOC) study | no | High-grade serous and endometrioid selection, selection for age of onset | - | - |
| Rahman  et al. 2018 | PMID: 29535157 | Mainstreamed genetic testing for women with ovarian cancer: first-year experience | no | High-grade non-mucinous tumour selection | - | - |
| Chiang  et al. 2006 | PMID: 16360812 | *BRCA1* promoter methylation predicts adverse ovarian cancer prognosis | no | HGSC vs other histopathology | - | - |
| Ramus  et al. 2001 | PMID: 19383375 | The contribution of *BRCA1* and *BRCA2* to ovarian cancer | no | HGSC vs other histopathology | - | - |
| You  et al. 2020 | [PMID: 32211327](https://pubmed.ncbi.nlm.nih.gov/32211327) | Germline and Somatic *BRCA1/2* Mutations in 172 Chinese Women with Epithelial Ovarian Cancer | no | HGSC vs non-HGSC counts | - | - |
| Ben David  et al. 2019 | PMID: 11786575 | Effect of *BRCA* mutations on the length of survival in epithelial ovarian tumors | no | HGSC histopathology selection | - | - |
| Cass  et al. 2003 | PMID: 12712470 | Improved survival in women with *BRCA*-associated ovarian carcinoma | no | HGSC histopathology selection | - | - |
| Gelmon  et al. 2011 | PMID: 21862407 | Olaparib in patients with recurrent high-grade serous or poorly differentiated ovarian carcinoma or triple-negative breast cancer: a phase 2, multicentre, open-label, non-randomised study | no | HGSC histopathology selection | - | - |
| Hyman  et al. 2012 | [PMID: 22139894](https://pubmed.ncbi.nlm.nih.gov/22139894) | Improved survival for *BRCA2*-associated serous ovarian cancer compared with both *BRCA*-negative and *BRCA1*-associated serous ovarian cancer | no | HGSC histopathology selection | - | - |
| Ledderman et al. 2012 | [PMID: 27824811](https://pubmed.ncbi.nlm.nih.gov/27824811) | Quality of life during olaparib maintenance therapy in platinum-sensitive relapsed serous ovarian cancer | no | HGSC histopathology selection | - | - |
| Yang  et al. 2011 | PMID: 21990299 | Association of *BRCA1* and *BRCA2* mutations with survival, chemotherapy sensitivity, and gene mutator phenotype in patients with ovarian cancer | no | HGSC histopathology selection | - | - |
| Manchana et al. 2019 | [PMID: 31467961](https://pubmed.ncbi.nlm.nih.gov/31467961) | *BRCA* mutation in high grade epithelial ovarian cancers | no | HGSC and high-grade endometrioid histopathology selection | - | - |
| Mehta  et al. 2018 | PMID: 30555256 | Germline *BRCA1* and *BRCA2* deleterious mutations and variants of unknown clinical significance associated with breast/ovarian cancer: a report from North India | no | Does not distinguish between VUS and PV | - | - |
| Rumford  et al. 2020 | PMID: 32098980 | Oncologist-led *BRCA* 'mainstreaming' in the ovarian cancer clinic: A study of 255 patients and its impact on their management | no | >90% HGSC | - | - |
| Stratton  et al. 1997 | PMID: 9099656 | Contribution of *BRCA1* mutations to ovarian cancer | no | All serous are of unknown grade | - | *BRCA1* data only |
| Rust  et al. 2018 | PMID: 29460478 | Routine germline *BRCA1* and *BRCA2* testing in patients with ovarian carcinoma: analysis of the Scottish real-life experience | no | Non-mucinous selection | - | Contains mucinous |
| Tan  et al. 2008 | PMID: 18955455 | "*BRCA*ness" syndrome in ovarian cancer: a case-control study describing the clinical features and outcome of patients with epithelial ovarian cancer associated with *BRCA1* and *BRCA2* mutations | no | No differentiation between HGSC & LGSC | - | Data include samples from Stratton et al. 2012 |
| Alsop  et al. 2012 | [PMID: 22711857](https://pubmed.ncbi.nlm.nih.gov/22711857) | *BRCA* mutation frequency and patterns of treatment response in *BRCA* mutation-positive women with ovarian cancer: a report from the Australian Ovarian Cancer Study Group | no | Non-mucinous selection, No differentiation between HGSC & LGSC | - | Non-borderline phenotype selection |
| Schrader  et al. 2012 | PMID: 22776961 | Germline *BRCA1* and *BRCA2* mutations in ovarian cancer: utility of a histology-based referral strategy | no | Non-mucinous selection | - | Non-borderline phenotype selection |
| McLaughlin  et al. 2012 | [PMID: 23257159](https://pubmed.ncbi.nlm.nih.gov/23257159) | Long-term ovarian cancer survival associated with mutation in *BRCA1* or *BRCA2* | no | No differentiation between HGSC & LGSC | - | Non-borderline phenotype selection |
| Tonin  et al. 2007 | PMID: 17636423 | A review of histopathological subtypes of ovarian cancer in *BRCA*-related French Canadian cancer families | no | No differentiation between HGSC & LGSC | - | Non-borderline phenotype selection |
| Hauke e  t al. 2019 | PMID: 30979843 | Deleterious somatic variants in 473 consecutive individuals with ovarian cancer: results of the observational AGO-TR1 study (NCT02222883) | no | Non-mucinous selection | - | Non-borderline phenotype selection, 'Other' samples unspecified excluded |
| Norquist  et al. 2013 | PMID: 23262210 | Characteristics of women with ovarian carcinoma who have *BRCA1* and *BRCA2* mutations not identified by clinical testing | no | Potential overlap with Norquist et al. 2016, Joined *BRCA1/2* data | - |  |
| Candido-dos Reis  et al. 2015 | PMID: 25398451 | Germline mutation in *BRCA1* or *BRCA2* and ten-year survival for women diagnosed with epithelial ovarian cancer | no | Potential overlap with CIMBA | - |  |

HGSC; High-grade serous carcinoma, LGSC; Low-grade serous carcinoma, VUS; Variants of uncertain significance, PV; Pathogenic variant

**Supplementary Table S2**

**Ovarian cancer case data collected from relevant sources according to histology subtype and carrier status**

|  | *BRCA1* carriers | | | | | | *BRCA2* carriers | | | | | | Non-carriers | | | | | |
| --- | --- | --- | --- | --- | --- | --- | --- | --- | --- | --- | --- | --- | --- | --- | --- | --- | --- | --- |
| Data source | HGSC | LGSC | Muc | End | CC | ‘Other’ | HGSC | LGSC | Muc | End | CC | ‘Other’ | HGSC | LGSC | Muc | End | CC | ‘Other’ |
| CIMBA consortium |  |  |  |  |  |  |  |  |  |  |  |  |  |  |  |  |  |  |
| BCFR-NY | 5 | 0 | 0 | 1 | 1 | 0 | 2 | 0 | 1 | 2 | 0 | 1 | 0 | 0 | 0 | 0 | 0 | 0 |
| BCFR-ON | 0 | 0 | 0 | 1 | 0 | 0 | 0 | 0 | 0 | 0 | 0 | 0 | 0 | 0 | 0 | 0 | 0 | 0 |
| BCFR-PA | 1 | 0 | 0 | 1 | 0 | 0 | 1 | 0 | 0 | 0 | 0 | 0 | 0 | 0 | 0 | 0 | 0 | 0 |
| BRICOH | 10 | 0 | 0 | 5 | 0 | 3 | 3 | 1 | 0 | 1 | 0 | 3 | 0 | 0 | 0 | 0 | 0 | 0 |
| CBCS | 10 | 0 | 0 | 1 | 0 | 1 | 3 | 0 | 0 | 1 | 0 | 0 | 0 | 0 | 0 | 0 | 0 | 0 |
| CNIO | 0 | 0 | 1 | 0 | 0 | 0 | 2 | 0 | 0 | 3 | 0 | 0 | 0 | 0 | 0 | 0 | 0 | 0 |
| CCGCRN | 0 | 0 | 0 | 1 | 0 | 0 | 0 | 0 | 0 | 0 | 0 | 0 | 0 | 0 | 0 | 0 | 0 | 0 |
| CONSIT TEAM | 86 | 0 | 1 | 33 | 3 | 20 | 26 | 2 | 0 | 2 | 0 | 3 | 0 | 0 | 0 | 0 | 0 | 0 |
| DEMOKRITOS | 27 | 0 | 1 | 9 | 1 | 0 | 0 | 0 | 0 | 0 | 0 | 0 | 0 | 0 | 0 | 0 | 0 | 0 |
| EMBRACE | 97 | 2 | 6 | 27 | 5 | 7 | 60 | 3 | 2 | 10 | 3 | 6 | 0 | 0 | 0 | 0 | 0 | 0 |
| FCCC | 5 | 0 | 0 | 0 | 0 | 0 | 2 | 0 | 0 | 0 | 0 | 0 | 0 | 0 | 0 | 0 | 0 | 0 |
| FPGMX | 18 | 1 | 0 | 2 | 1 | 0 | 3 | 0 | 0 | 0 | 0 | 0 | 0 | 0 | 0 | 0 | 0 | 0 |
| G-FAST | 5 | 0 | 1 | 2 | 0 | 3 | 1 | 0 | 0 | 0 | 0 | 0 | 0 | 0 | 0 | 0 | 0 | 0 |
| GC-HBOC | 94 | 3 | 1 | 10 | 0 | 0 | 19 | 0 | 1 | 2 | 0 | 0 | 0 | 0 | 0 | 0 | 0 | 0 |
| GEMO | 43 | 8 | 0 | 15 | 1 | 3 | 10 | 3 | 3 | 5 | 0 | 1 | 0 | 0 | 0 | 0 | 0 | 0 |
| HCSC | 6 | 1 | 0 | 3 | 0 | 0 | 5 | 0 | 0 | 0 | 0 | 1 | 0 | 0 | 0 | 0 | 0 | 0 |
| HEBCS | 1 | 0 | 0 | 9 | 0 | 1 | 2 | 0 | 0 | 2 | 0 | 3 | 0 | 0 | 0 | 0 | 0 | 0 |
| HEBON | 8 | 1 | 0 | 1 | 0 | 0 | 3 | 0 | 2 | 0 | 0 | 0 | 0 | 0 | 0 | 0 | 0 | 0 |
| ICO | 9 | 0 | 0 | 1 | 0 | 3 | 3 | 0 | 0 | 1 | 0 | 1 | 0 | 0 | 0 | 0 | 0 | 0 |
| IHCC | 2 | 0 | 0 | 0 | 0 | 1 | 0 | 0 | 0 | 0 | 0 | 0 | 0 | 0 | 0 | 0 | 0 | 0 |
| ILUH | 0 | 0 | 0 | 0 | 0 | 0 | 2 | 0 | 0 | 1 | 0 | 0 | 0 | 0 | 0 | 0 | 0 | 0 |
| INHERIT | 0 | 0 | 0 | 1 | 0 | 0 | 1 | 0 | 1 | 0 | 0 | 0 | 0 | 0 | 0 | 0 | 0 | 0 |
| IOVHBOCS | 34 | 1 | 0 | 4 | 0 | 2 | 10 | 0 | 0 | 1 | 0 | 1 | 0 | 0 | 0 | 0 | 0 | 0 |
| IPOBCS | 7 | 1 | 0 | 2 | 2 | 2 | 2 | 0 | 0 | 1 | 0 | 0 | 0 | 0 | 0 | 0 | 0 | 0 |
| KCONFAB | 37 | 1 | 2 | 4 | 0 | 3 | 10 | 0 | 0 | 1 | 0 | 0 | 0 | 0 | 0 | 0 | 0 | 0 |
| KUMC | 1 | 0 | 0 | 0 | 0 | 0 | 0 | 0 | 0 | 0 | 0 | 0 | 0 | 0 | 0 | 0 | 0 | 0 |
| MSKCC | 27 | 2 | 0 | 1 | 1 | 0 | 0 | 0 | 0 | 2 | 0 | 0 | 0 | 0 | 0 | 0 | 0 | 0 |
| MUV | 29 | 0 | 0 | 1 | 0 | 4 | 1 | 0 | 0 | 1 | 0 | 0 | 0 | 0 | 0 | 0 | 0 | 0 |
| NORTHSHORE | 9 | 0 | 0 | 1 | 0 | 0 | 3 | 0 | 0 | 1 | 1 | 0 | 0 | 0 | 0 | 0 | 0 | 0 |
| OSU CCG | 2 | 0 | 0 | 1 | 0 | 1 | 3 | 0 | 0 | 1 | 1 | 0 | 0 | 0 | 0 | 0 | 0 | 0 |
| OUH | 4 | 0 | 0 | 0 | 0 | 1 | 3 | 0 | 0 | 1 | 0 | 1 | 0 | 0 | 0 | 0 | 0 | 0 |
| PBCS | 4 | 1 | 0 | 0 | 0 | 5 | 0 | 0 | 0 | 0 | 0 | 0 | 0 | 0 | 0 | 0 | 0 | 0 |
| SWE-BRCA | 8 | 1 | 0 | 7 | 0 | 0 | 0 | 0 | 0 | 0 | 0 | 0 | 0 | 0 | 0 | 0 | 0 | 0 |
| UCSF | 19 | 0 | 0 | 2 | 0 | 1 | 6 | 0 | 0 | 0 | 0 | 1 | 0 | 0 | 0 | 0 | 0 | 0 |
| UKGRFOCR | 8 | 1 | 0 | 3 | 0 | 16 | 3 | 0 | 0 | 2 | 0 | 4 | 0 | 0 | 0 | 0 | 0 | 0 |
| UPENN | 0 | 0 | 1 | 4 | 0 | 2 | 0 | 0 | 0 | 1 | 0 | 0 | 0 | 0 | 0 | 0 | 0 | 0 |
| UPITT | 10 | 0 | 0 | 2 | 0 | 0 | 0 | 0 | 0 | 0 | 0 | 0 | 0 | 0 | 0 | 0 | 0 | 0 |
| VFCTG | 20 | 0 | 1 | 3 | 0 | 1 | 7 | 0 | 1 | 0 | 0 | 3 | 0 | 0 | 0 | 0 | 0 | 0 |
| WCP | 49 | 2 | 0 | 1 | 0 | 2 | 16 | 1 | 0 | 1 | 0 | 0 | 0 | 0 | 0 | 0 | 0 | 0 |
| OTTA consortium |  |  |  |  |  |  |  |  |  |  |  |  |  |  |  |  |  |  |
| ORE | 7 | 0 | 0 | 0 | 0 | 0 | 4 | 0 | 0 | 1 | 0 | 0 | 28 | 0 | 2 | 5 | 3 | 0 |
| SEA | 19 | 0 | 0 | 0 | 1 | 0 | 24 | 0 | 0 | 2 | 0 | 1 | 271 | 17 | 40 | 101 | 74 | 37 |
| STA | 25 | 1 | 0 | 1 | 2 | 1 | 1 | 0 | 0 | 0 | 0 | 0 | 185 | 16 | 47 | 40 | 28 | 16 |
| Individual sites |  |  |  |  |  |  |  |  |  |  |  |  |  |  |  |  |  |  |
| Brazil | 46 | 1 | 0 | 4 | 1 | 3 | 10 | 0 | 0 | 1 | 0 | 1 | 138 | 9 | 8 | 8 | 12 | 15 |
| CTE | 44 | 0 | 0 | 4 | 0 | 0 | 17 | 0 | 0 | 1 | 1 | 1 | 178 | 16 | 13 | 48 | 25 | 37 |
| IRST | 7 | 0 | 0 | 0 | 0 | 0 | 7 | 0 | 0 | 1 | 0 | 0 | 16 | 1 | 0 | 5 | 3 | 1 |
| MUV | 62 | 8 | 0 | 2 | 0 | 2 | 17 | 5 | 0 | 1 | 0 | 1 | 196 | 13 | 12 | 35 | 9 | 11 |
| HJO | 24 | 0 | 0 | 2 | 0 | 3 | 12 | 0 | 0 | 1 | 1 | 5 | 133 | 26 | 23 | 18 | 2 | 1 |
| DEMOKRITOS | 117 | 2 | 1 | 5 | 4 | 4 | 41 | 0 | 0 | 1 | 0 | 2 | 330 | 20 | 10 | 50 | 22 | 18 |
| CNIO | 30 | 4 | 2 | 6 | 1 | 5 | 11 | 2 | 0 | 2 | 1 | 3 | 14 | 6 | 3 | 3 | 3 | 4 |
| CZECANCA | 124 | 4 | 2 | 17 | 0 | 11 | 48 | 4 | 2 | 3 | 0 | 0 | 328 | 78 | 53 | 72 | 14 | 28 |
| *Total research or clinical data* | 1,200 | 46 | 20 | 200 | 24 | 111 | 404 | 21 | 13 | 57 | 8 | 43 | 1,817 | 202 | 211 | 385 | 195 | 168 |
| Literature |  |  |  |  |  |  |  |  |  |  |  |  |  |  |  |  |  |  |
| Atavesen 2020 | 0 | 0 | 0 | 0 | 0 | 0 | 0 | 0 | 0 | 0 | 0 | 0 | 310 | 33 | 16 | 28 | 23 | 8 |
| Bernards 2016 | 6 | 0 | 0 | 2 | 1 | 1 | 1 | 0 | 0 | 0 | 0 | 0 | 14 | 7 | 3 | 7 | 4 | 1 |
| Brozek 2008 | 9 | 0 | 0 | 0 | 3 | 1 | 8 | 0 | 0 | 0 | 0 | 0 | 0 | 0 | 0 | 0 | 0 | 0 |
| Bu 2019 | 0 | 0 | 0 | 0 | 0 | 0 | 0 | 0 | 0 | 0 | 0 | 0 | 301 | 27 | 11 | 16 | 16 | 18 |
| Enomoto 2020 | 53 | 1 | 0 | 7 | 2 | 0 | 25 | 0 | 0 | 1 | 2 | 0 | 181 | 4 | 19 | 104 | 178 | 28 |
| Hilton 2002 | 0 | 0 | 0 | 0 | 0 | 0 | 5 | 0 | 0 | 0 | 0 | 0 | 0 | 0 | 0 | 0 | 0 | 0 |
| Hirasawa 2017 | 15 | 0 | 0 | 1 | 1 | 1 | 7 | 0 | 0 | 1 | 1 | 0 | 8 | 0 | 1 | 2 | 2 | 1 |
| Johansson 1998 | 13 | 1 | 1 | 7 | 0 | 0 | 0 | 0 | 0 | 0 | 0 | 0 | 0 | 0 | 0 | 0 | 0 | 0 |
| Khoo 2000 | 5 | 0 | 0 | 1 | 0 | 0 | 0 | 0 | 1 | 0 | 0 | 0 | 0 | 0 | 0 | 0 | 0 | 0 |
| Kim 2020 | 0 | 0 | 0 | 0 | 0 | 0 | 0 | 0 | 0 | 0 | 0 | 0 | 246 | 10 | 13 | 37 | 40 | 16 |
| Kowalik 2019 | 20 | 5 | 0 | 1 | 1 | 0 | 7 | 1 | 0 | 0 | 0 | 0 | 0 | 0 | 0 | 0 | 0 | 0 |
| Li 2019 | 12 | 0 | 0 | 0 | 0 | 0 | 2 | 0 | 0 | 0 | 0 | 0 | 35 | 0 | 1 | 3 | 4 | 3 |
| Norquist 2016 | 155 | 3 | 0 | 4 | 4 | 2 | 85 | 1 | 0 | 3 | 0 | 0 | 1,261 | 66 | 16 | 71 | 54 | 29 |
| Sakamoto 2015 | 5 | 0 | 0 | 0 | 0 | 0 | 7 | 0 | 0 | 0 | 0 | 0 | 45 | 17 | 0 | 6 | 10 | 5 |
| Sarantaus 2001 | 1 | 0 | 0 | 0 | 0 | 4 | 1 | 0 | 0 | 1 | 1 | 1 | 1 | 1 | 0 | 1 | 0 | 1 |
| Seo 2019 | 53 | 2 | 0 | 1 | 0 | 1 | 31 | 0 | 0 | 0 | 0 | 0 | 392 | 30 | 10 | 17 | 27 | 9 |
| Soegaard 2008 | 13 | 0 | 0 | 2 | 2 | 1 | 1 | 0 | 0 | 1 | 1 | 0 | 0 | 0 | 0 | 0 | 0 | 0 |
| Sugino 2019 | 6 | 0 | 0 | 0 | 0 | 0 | 4 | 0 | 0 | 1 | 1 | 1 | 2 | 1 | 0 | 1 | 0 | 7 |
| Vos 2020 | 12 | 0 | 0 | 0 | 0 | 1 | 9 | 0 | 0 | 0 | 1 | 2 | 155 | 16 | 16 | 12 | 18 | 15 |
| Wu 2017 | 0 | 0 | 0 | 0 | 0 | 0 | 0 | 0 | 0 | 0 | 0 | 0 | 415 | 15 | 8 | 23 | 34 | 4 |
| *Total literature* | 378 | 12 | 1 | 26 | 14 | 12 | 193 | 2 | 1 | 8 | 7 | 4 | 3,366 | 227 | 114 | 328 | 410 | 145 |
| *Total data from all sources* | **1,578** | **58** | **21** | **226** | **38** | **123** | **597** | **23** | **14** | **65** | **15** | **47** | **5,183** | **429** | **325** | **713** | **605** | **313** |

HGSC; High-grade serous carcinoma, LGSC; Low-grade serous carcinoma, Muc; Mucinous, End; Endometrioid, CC; Clear cell. The ‘other’ category denominates rare forms of ovarian cancer not belonging to any of the other subtypes, including tumours defined as: ‘other’ by data sources not specifying tumour histology; mixed-epithelial; carcinosarcomas; transitional cell (Brenner tumours); undifferentiated or poorly differentiated; squamous cell.

**Supplementary Table S3**

**Characterisation of ovarian tumour histotypes according to tumour stage, grade and age at ovarian cancer diagnosis**

|  | | FIGO stage | | | | |  | Tumour grade | | | |  | Age at ovarian  cancer diagnosis |
| --- | --- | --- | --- | --- | --- | --- | --- | --- | --- | --- | --- | --- | --- |
| Histotypes | N | Stage 1  N (%) | Stage 2  N (%) | Stage 3  N (%) | Stage 4  N (%) | Stage N/A N (%) | | Grade 1 N (%) | Grade 2 N (%) | Grade 3 N (%) | Grade N/A N (%) |  | Median  (Min-Max) |
| HGSC | 7,358 | 534 (7.3) | 759 (10.3) | 1,061 (14.4) | 297 (4.0) | 4,500 (61.2) | | N/A | N/A | N/A | N/A |  | 56 (20 - 92) |
| LGSC | 510 | 79 (15.5) | 39 (7.6) | 69 (13.5) | 10 (2.0) | 295 (57.8) | | N/A | N/A | N/A | N/A |  | 50 (18 - 84) |
| Mucinous | 360 | 150 (41.7) | 12 (3.3) | 15 (4.2) | 5 (1.4) | 167 (46.4) | | 56 (15.5) | 67 (18.6) | 35 (9.7) | 202 (56.1) |  | 55 (18 - 80) |
| Endometrioid | 1,004 | 271 (27.0) | 46 (4.6) | 94 (9.4) | 18 (1.8) | 537 (53.5) | | 131 (13.0) | 169 (16.8) | 234 (23.3) | 470 (46.8) |  | 51 (20 - 86) |
| Clear cell | 658 | 144 (21.9) | 19 (2.9) | 23 (3.5) | 5 (0.8) | 446 (67.8) | | 9 (1.4) | 23 (3.5) | 108 (16.4) | 518 (78.7) |  | 53 (23 - 85) |
| ‘Other’ | 483 | 85 (17.6) | 43 (8.9) | 59 (12.2) | 15 (3.1) | 254 (52.6) | | 14 (2.9) | 46 (9.5) | 164 (33.8) | 259 (53.6) |  | 55 (25 - 80) |
| *Total* | 10,373 |  |  |  |  | |  | |  |  |  |  |  |

N, Number of data points; HGSC, High-grade serous carcinomas; LGSC, Low-grade serous carcinomas; N/A, Not available; Min, minimum; Max, maximum
The above data are based on 10,373 cases, including 2,044 *BRCA1* carriers, 761 *BRCA2* carriers and 7,568 non-carriers. The ‘other’ category denominates rare forms of ovarian cancer not belonging to any of the other subtypes, including tumours defined as: ‘other’ by data sources not specifying tumour histology; mixed-epithelial; carcinosarcomas; transitional cell (Brenner tumours); undifferentiated or poorly differentiated; squamous cell. In brackets, the frequencies of different tumour stages and grades within each histotype category are provided. For HGSC and LGSC, grade differentiation is not applicable since the histotypes are by definition high-grade and low-grade respectively, according to a two-tier system.

**Supplementary Table S4**

**Clinical-tested or research-tested data and literature data likelihood ratio association analyses of ovarian tumour histotypes**

|  | *BRCA1* carriers | | | | | | | |  | | *BRCA2* carriers | | | | | | | | | |  | Non-carriers | | |  | | Total | |  |
| --- | --- | --- | --- | --- | --- | --- | --- | --- | --- | --- | --- | --- | --- | --- | --- | --- | --- | --- | --- | --- | --- | --- | --- | --- | --- | --- | --- | --- | --- |
| Histotype | N (%) | LR (95% CI) | | | ACMG/AMP strength | | | |  | | N (%) | | LR (95% CI) | | | | ACMG/AMP strength | | | |  | N (%) | | | |  | | N (%) | |
| *Clinical- or research-tested data* | | | |  | | | |  | | | |  | |  | |  | | | |  | | | |  |  |  |  |  |  |
| HGSC | 1,200 (75.0) | 1.23 (1.18-1.28) | | | non-informative | | | |  | | 404 (74.0) | | 1.21 (1.15-1.28) | | | | non-informative | | | |  | 1,817 (61.0) | | | |  | | 3,421 (66.8) | |
| LGSC | 46 (2.9) | **0.42 (0.31-0.58)** | | | **Supporting Benign** | | | |  | | 21 (3.8) | | 0.57 (0.37-0.88) | | | | non-informative | | | |  | 202 (6.8) | | | |  | | 269 (5.2) | |
| Mucinous | 20 (1.2) | **0.18 (0.11-0.28)** | | | **Moderate Benign** | | | |  | | 13 (2.4) | | **0.34 (0.19-0.58)** | | | | **Supporting Benign** | | | |  | 211 (7.1) | | | |  | | 244 (4.8) | |
| Endometrioid | 200 (12.5) | 0.97 (0.82-1.13) | | | non-informative | | | |  | | 57 (10.4) | | 0.81 (0.62-1.05) | | | | non-informative | | | |  | 385 (12.9) | | | |  | | 642 (12.5) | |
| Clear cell | 24 (1.5) | **0.23 (0.15-0.35)** | | | **Supporting Benign** | | | |  | | 8 (1.5) | | **0.22 (0.11-0.45)** | | | | **Moderate Benign** | | | |  | 195 (6.5) | | | |  | | 227 (4.4) | |
| ‘Other’ | 111 (6.9) | 1.23 (0.97-1.55) | | | non-informative | | | |  | | 43 (7.9) | | 1.40 (1.01-1.93) | | | | non-informative | | | |  | 168 (5.6) | | | |  | | 322 (6.3) | |
| Total | 1,601 |  | | |  | | | |  | | 546 | |  | | | |  | | | |  | 2,978 | | | |  | | 5,125 | |
| *Literature data* | | |  | | |  |  | | |  | | | | |  | | |  |  | | |  |  | | | |  |  |  |
| HGSC | 378 (85.3) | 1.16 (1.12-1.21) | | | non-informative | | | |  | | 193 (89.8) | | 1.22 (1.17-1.28) | | | | non-informative | | | |  | 3,366 (73.3) | | | |  | | 3,937 (75.0) | |
| LGSC | 12 (2.7) | 0.55 (0.31-0.97) | | | non-informative | | | |  | | 2 (0.9) | | **0.19 (0.05-0.75)** | | | | **Moderate Benign** | | | |  | 227 (4.9) | | | |  | | 241 (4.6) | |
| Mucinous | 1 (0.2) | **0.09 (0.01-0.65)** | | | **Moderate Benign** | | | |  | | 1 (0.5) | | 0.19 (0.03-1.33) | | | | Moderate Benign | | | |  | 114 (2.5) | | | |  | | 116 (2.2) | |
| Endometrioid | 26 (5.9) | 0.82 (0.56-1.21) | | | non-informative | | | |  | | 8 (3.7) | | 0.52 (0.26-1.04) | | | | non-informative | | | |  | 328 (7.1) | | | |  | | 362 (6.9) | |
| Clear cell | 14 (3.2) | **0.35 (0.21-0.60)** | | | **Supporting Benign** | | | |  | | 7 (3.3) | | **0.36 (0.17-0.76)** | | | | **Supporting Benign** | | | |  | 410 (8.9) | | | |  | | 431 (8.2) | |
| ‘Other’ | 12 (2.7) | 0.86 (0.48-1.53) | | | non-informative | | | |  | | 4 (1.9) | | 0.59 (0.22-1.58) | | | | non-informative | | | |  | 145 (3.2) | | | |  | | 161 (3.1) | |
| Total | 443 |  | | |  | | | |  | | 215 | |  | | | |  | | | |  | 4,590 | | | |  | | 5,248 | |
|  | 2,044 |  | | |  | | | |  | | 761 | |  | | | |  | | | |  | 7,571 | | | |  | | 10,373 | |

N, Number of data points; LR, Likelihood ratio; CI, Confidence Interval; ACMG/AMP, American College of Medical Genetics/Association for Molecular Pathology; HGSC, High-grade serous carcinomas; LGSC, Low-grade serous carcinomas. In brackets, the histotype frequency for each group is provided. The ‘other’ category denominates rare forms of ovarian cancer not belonging to any of the other subtypes, including tumours defined as: ‘other’ by data sources not specifying tumour histology; mixed-epithelial; carcinosarcomas; transitional cell (Brenner tumours); undifferentiated or poorly differentiated; squamous cell. LR>1: Histotype association with pathogenic variant, Pathogenic evidence; LR<1: Prediction of non-carrier for pathogenic variant, Benign evidence. Evidence strength was measured based on Bayesian modelling of ACMG/AMP rules (see Materials and Methods); Supporting Benign (LR≥0.23-0.48), Moderate Benign (LR≥0.053-0.23), Supporting Pathogenic (LR≥2.08-4.30), non-informative (0.48≤LR≤2.08). LR estimates reaching informative ACMG/AMP strengths at a statistically significant CI (i.e., not spanning 1), are highlighted in bold.

**Supplementary Table S5**

**European- and Asian-origin ancestry likelihood ratio association analyses of ovarian tumour histotypes**

|  |  | *BRCA1* carriers | | |  | |  | | *BRCA2* carriers | | | |  | |  | | Non-carriers | |  | | Total | |
| --- | --- | --- | --- | --- | --- | --- | --- | --- | --- | --- | --- | --- | --- | --- | --- | --- | --- | --- | --- | --- | --- | --- |
| Histotype |  | N (%) | LR (95% CI) | | ACMG/AMP strength | |  | | N (%) | | LR (95% CI) | | ACMG/AMP strength | |  | | N (%) | |  | | N (%) | |
| *Asian-origin ancestry* | | | |  | |  | |  | |  | |  | |  | |  | |  | |  | |  |
| HGSC |  | 151 (89.3) | 1.33 (1.26-1.41) | | non-informative | |  | | 77 (89.5) | | 1.34 (1.24-1.44) | | non-informative | |  | | 1,634 (67.0) | |  | | 1,862 (69.1) | |
| LGSC |  | 3 (1.8) | 0.41 (0.13-1.29) | | Supporting Benign | |  | | 0 (0.0) | | - | | - | |  | | 105 (4.3) | |  | | 108 (4.0) | |
| Mucinous |  | 0 (0.0) | - | | - | |  | | 1 (1.2) | | 0.41 (0.06-2.88) | | Supporting Benign | |  | | 70 (2.9) | |  | | 71 (2.6) | |
| Endometrioid |  | 10 (5.9) | 0.66 (0.36-1.22) | | non-informative | |  | | 3 (3.5) | | 0.39 (0.13-1.19) | | Supporting Benign | |  | | 219 (9.0) | |  | | 232 (8.6) | |
| Clear cell |  | 3 (1.8) | **0.14 (0.04-0.42)** | | **Moderate Benign** | |  | | 4 (4.7) | | **0.36 (0.14-0.93)** | | **Supporting Benign** | |  | | 318 (13.0) | |  | | 325 (12.1) | |
| ‘Other' |  | 2 (1.2) | 0.31 (0.08-1.25) | | Supporting Benign | |  | | 1 (1.2) | | 0.30 (0.04-2.16) | | Supporting Benign | |  | | 93 (3.8) | |  | | 96 (3.6) | |
| Total |  | 169 |  | |  | |  | | 86 | |  | |  | |  | | 2,439 | |  | | 2,694 | |
| *European-origin ancestry* | | | |  | |  | |  | |  | |  | |  | |  | |  | |  | |  |
| HGSC |  | 1,215 (74.4) | 1.17 (1.13-1.22) | | non-informative | |  | | 418 (74.1) | | 1.17 (1.11-1.24) | | non-informative | |  | | 2,160 (63.3) | |  | | 3,793 (67.6) | |
| LGSC |  | 51 (3.1) | **0.45 (0.33-0.60)** | | **Supporting Benign** | |  | | 22 (3.9) | | 0.56 (0.37-0.86) | | non-informative | |  | | 237 (6.9) | |  | | 310 (5.5) | |
| Mucinous |  | 21 (1.3) | **0.20 (0.13-0.31)** | | **Moderate Benign** | |  | | 13 (2.3) | | **0.35 (0.20-0.62)** | | **Supporting Benign** | |  | | 222 (6.5) | |  | | 256 (4.6) | |
| Endometrioid |  | 206 (12.6) | 1.06 (0.91-1.24) | | non-informative | |  | | 57 (10.1) | | 0.85 (0.65-1.11) | | non-informative | |  | | 405 (11.9) | |  | | 668 (11.9) | |
| Clear cell |  | 29 (1.8) | **0.28 (0.19-0.42)** | | **Supporting Benign** | |  | | 10 (1.8) | | **0.28 (0.15-0.53)** | | **Supporting Benign** | |  | | 214 (6.3) | |  | | 253 (4.5) | |
| ‘Other' |  | 111 (6.8) | 1.34 (1.06-1.69) | | non-informative | |  | | 44 (7.8) | | 1.54 (1.12-2.12) | | non-informative | |  | | 173 (5.1) | |  | | 328 (5.8) | |
| Total |  | 1,633 |  | |  | |  | | 564 | |  | |  | |  | | 3,411 | |  | | 5,608 | |
|  |  | 1,802 |  | |  | |  | | 650 | |  | |  | |  | | 5,850 | |  | | 8,302 | |

N, Number of data points; LR, Likelihood ratio; CI, Confidence Interval; ACMG/AMP, American College of Medical Genetics/Association for Molecular Pathology; HGSC, High-grade serous carcinomas; LGSC, Low-grade serous carcinomas. European-origin ancestry category comprises of European, Oceanian and North American descent. LR estimates for Hispanic and African ancestries were non-informative. The ‘other’ category denominates rare forms of ovarian cancer not belonging to any of the other subtypes, including tumours defined as: ‘other’ by data sources not specifying tumour histology; mixed-epithelial; carcinosarcomas; transitional cell (Brenner tumours); undifferentiated or poorly differentiated; squamous cell. In brackets, the histotype frequency for each group is provided. LR>1: Histotype association with pathogenic variant, Pathogenic evidence; LR<1: Prediction of non-carrier for pathogenic variant, Benign evidence. Evidence strength was measured based on Bayesian modelling of ACMG/AMP rules (see Materials and Methods); Supporting Benign (LR≥0.23-0.48), Moderate Benign (LR≥0.053-0.23), Supporting Pathogenic (LR≥2.08-4.30), non-informative (0.48≤LR≤2.08). LR estimates reaching informative ACMG/AMP strengths at a statistically significant CI (i.e., not spanning 1), are highlighted in bold.

**Supplementary Table S6**

**Likelihood ratio association analysis based on ovarian tumour histology and tumour grade**

| Tumour Pathology | |  | *BRCA1* carriers | |  | |  | | *BRCA2* carriers | | |  | | | |  | | | Non-carriers | | |  | | | Total | | |
| --- | --- | --- | --- | --- | --- | --- | --- | --- | --- | --- | --- | --- | --- | --- | --- | --- | --- | --- | --- | --- | --- | --- | --- | --- | --- | --- | --- |
| Histotype | Grade |  | N (%) | LR (95% CI) | ACMG/AMP strength | |  | | N (%) | LR (95% CI) | ACMG/AMP strength | | | |  | | | N (%) | | |  | | | N (%) | | |  |
| Serous |  |  |  |  |  | |  | |  |  |  | | | |  | | |  | | |  | | |  | | |  |
|  | High-grade |  | 1,578 (82.7) | 1.09 (1.06-1.11) | non-informative |  | | 597 (83.6) | | 1.10 (1.06-1.14) | | | non-informative |  | | | 5,183 (76.0) | | |  | | | 7,358 (77.9) | | |  |  |
|  | Low-grade |  | 58 (3.0) | 0.48 (0.37-0.63) | non-informative |  | | 23 (3.2) | | 0.51 (0.34-0.77) | | | non-informative |  | | | 429 (6.3) | | |  | | | 510 (5.4) | | |  |  |
| Mucinous |  |  |  |  |  |  | |  | |  | | |  |  | | |  | | |  | | |  | | |  |  |
|  | Grade 1 |  | 1 (0.1) | **0.07 (0.01-0.50)** | **Moderate Benign** |  | | 2 (0.3) | | 0.37 (0.09-1.50) | | | Supporting Benign |  | | | 52 (0.8) | | |  | | | 55 (0.6) | | |  |  |
|  | Grade 2 |  | 5 (0.3) | **0.31 (0.12-0.77)** | **Supporting Benign** |  | | 4 (0.6) | | 0.66 (0.24-1.81) | | | non-informative |  | | | 58 (0.9) | | |  | | | 67 (0.7) | | |  |  |
|  | Grade 3 |  | 4 (0.2) | 0.55 (0.19-1.57) | non-informative |  | | 5 (0.7) | | 1.84 (0.71-4.77) | | | non-informative |  | | | 26 (0.4) | | |  | | | 35 (0.4) | | |  |  |
| Endometrioid |  |  |  |  |  |  | |  | |  | | |  |  | | |  | | |  | | |  | | |  |  |
|  | Grade 1 |  | 4 (0.2) | **0.12 (0.04-0.31)** | **Moderate Benign** |  | | 4 (0.6) | | **0.31 (0.12-0.84)** | | | **Supporting Benign** |  | | | 123 (1.8) | | |  | | | 131 (1.4) | | |  |  |
|  | Grade 2 |  | 40 (2.1) | 1.19 (0.84-1.70) | non-informative |  | | 9 (1.3) | | 0.72 (0.37-1.40) | | | non-informative |  | | | 120 (1.8) | | |  | | | 169 (1.8) | | |  |  |
|  | Grade 3 |  | 95 (5.0) | **2.98 (2.28-3.89)** | **Supporting Pathogenic** |  | | 25 (3.5) | | **2.09 (1.37-3.21)** | | | **Supporting Pathogenic** |  | | | 114 (1.7) | | |  | | | 234 (2.5) | | |  |  |
| ‘Other’ |  |  |  |  |  |  | |  | |  | | |  |  | | |  | | |  | | |  | | |  |  |
|  | Grade 1 |  | 3 (0.2) | 1.19 (0.32-4.39) | non-informative |  | | 2 (0.3) | | 2.12 (0.46-9.80) | | | Supporting Pathogenic |  | | | 9 (0.1) | | |  | | | 14 (0.1) | | |  |  |
|  | Grade 2 |  | 13 (0.7) | 1.50 (0.79-2.86) | non-informative |  | | 3 (0.4) | | 0.92 (0.28-3.02) | | | non-informative |  | | | 31 (0.5) | | |  | | | 47 (0.5) | | |  |  |
|  | Grade 3 |  | 70 (3.7) | **3.62 (2.61-5.03)** | **Supporting Pathogenic** |  | | 25 (3.5) | | **3.46 (2.20-5.43)** | | | **Supporting Pathogenic** |  | | | 69 (1.0) | | |  | | | 164 (1.7) | | |  |  |
|  | Total |  | 2,044 |  |  | |  | | 761 |  |  | | | |  | | | 7,570 | | |  | | | 10,373 | | |  |

N, Number of data points; LR, Likelihood ratio; CI, Confidence Interval; ACMG/AMP, American College of Medical Genetics/Association for Molecular Pathology. Serous tumours were categorised as high- and low-grade. Mucinous, endometrioid and ‘other’ histotypes were categorised into grade 1 (well-differentiated), grade 2 (moderately-differentiated) and grade 3 (undifferentiated or poorly-differentiated). By definition the clear cell histology is high-grade and therefore, separation of this subtype according to grade was not performed. The resulting distribution and total number of data considered in the above analysis also include data of clear cell histology and tumours of unknown grade information. The ‘other’ category denominates rare forms of ovarian cancer not belonging to any of the other subtypes, including tumours defined as: ‘other’ by data sources not specifying tumour histology; mixed-epithelial; carcinosarcomas; transitional cell (Brenner tumours); undifferentiated or poorly differentiated; squamous cell. In brackets, the histotype frequency for each group is provided. LR>1: Histotype association with pathogenic variant, Pathogenic evidence; LR<1: Prediction of non-carrier for pathogenic variant, Benign evidence. Evidence strength was measured based on Bayesian modelling of ACMG/AMP rules (see Materials and Methods); Supporting Benign (LR≥0.23-0.48), Moderate Benign (LR≥0.053-0.23), Supporting Pathogenic (LR≥2.08-4.30), non-informative (0.48≤LR≤2.08). LR estimates reaching informative ACMG/AMP strengths at a statistically significant CI (i.e., not spanning 1), are highlighted in bold.

**Supplementary Table S7**

**Likelihood ratio association analysis based on ovarian tumour invasion and tumour histology**

| Tumour Pathology | |  | *BRCA1* carriers | | | |  | | *BRCA2* carriers | | |  | Non-carriers |  | Total |
| --- | --- | --- | --- | --- | --- | --- | --- | --- | --- | --- | --- | --- | --- | --- | --- |
| Tumour invasion | Histotype |  | N (%) | LR (95% CI) | ACMG/AMP strength |  | | N (%) | | LR (95% CI) | ACMG/AMP strength |  | N (%) |  | N (%) |
| Invasive |  |  |  |  |  |  | |  | |  |  |  |  |  |  |
|  | HGSC |  | 1,062 (74.5) | 1.24 (1.19-1.29) | non-informative |  | | 375 (73.8) | | 1.23 (1.16-1.30) | non-informative |  | 1,746 (60.1) |  | 3,183 (65.8) |
|  | LGSC |  | 38 (2.7) | **0.44 (0.31-0.62)** | **Supporting Benign** |  | | 21 (4.1) | | 0.69 (0.44-1.07) | non-informative |  | 175 (6.0) |  | 234 (4.8) |
|  | Mucinous |  | 16 (1.1) | **0.22 (0.13-0.37)** | **Moderate Benign** |  | | 10 (2.0) | | **0.39 (0.21-0.73)** | **Supporting Benign** |  | 147 (5.1) |  | 173 (3.6) |
|  | Endometrioid |  | 181 (12.7) | 1.04 (0.88-1.23) | non-informative |  | | 50 (9.8) | | 0.81 (0.61-1.07) | non-informative |  | 354 (12.2) |  | 585 (12.1) |
|  | Clear cell |  | 21 (1.5) | **0.22 (0.14-0.35)** | **Moderate Benign** |  | | 7 (1.4) | | **0.21 (0.10-0.44)** | **Moderate Benign** |  | 192 (6.6) |  | 220 (4.5) |
|  | ‘Other’ |  | 101 (7.1) | 1.50 (1.17-1.93) | non-informative |  | | 40 (7.9) | | 1.67 (1.19-2.34) | non-informative |  | 137 (4.7) |  | 278 (5.7) |
| Borderline |  |  | 7 (0.5) | **0.09 (0.04-0.20)** | **Moderate Benign** |  | | 5 (1.0) | | **0.19 (0.08-0.46)** | **Moderate Benign** |  | 152 (5.2) |  | 164 (3.4) |
|  | Total |  | 1,426 |  |  |  | | 508 | |  |  |  | 2,903 |  | 4,837 |

N, Number of data points; LR, Likelihood ratio; CI, Confidence Interval; ACMG/AMP, American College of Medical Genetics/Association for Molecular Pathology; HGSC, High-grade serous carcinomas; LGSC, Low-grade serous carcinomas. Borderline tumours were considered separately to the main histotypes which were strictly invasive. Published studies applying sample selection based on tumour invasiveness were excluded. The ‘other’ category denominates rare forms of ovarian cancer not belonging to any of the other subtypes, including tumours defined as: ‘other’ by data sources not specifying tumour histology; mixed-epithelial; carcinosarcomas; transitional cell (Brenner tumours); undifferentiated or poorly differentiated; squamous cell. In brackets, the histotype frequency for each group is provided. LR>1: Histotype association with pathogenic variant, Pathogenic evidence; LR<1: Prediction of non-carrier for pathogenic variant, Benign evidence. Evidence strength was measured based on Bayesian modelling of ACMG/AMP rules (see Materials and Methods); Supporting Benign (LR≥0.23-0.48), Moderate Benign (LR≥0.053-0.23), non-informative (0.48≤LR≤2.08). LR estimates reaching informative ACMG/AMP strengths at a statistically significant CI (i.e., not spanning 1), are highlighted in bold.

**Supplementary Table S8**

**Likelihood ratio association analysis based on ovarian tumour histology and age at diagnosis**

| Tumour/patient characteristics | |  | *BRCA1* carriers | | |  | *BRCA2* carriers | | |  | Non-carriers |  | Total |
| --- | --- | --- | --- | --- | --- | --- | --- | --- | --- | --- | --- | --- | --- |
| Age at  diagnosis | Histotype |  | N (%) | LR (95% CI) | ACMG/AMP strength |  | N (%) | LR (95% CI) | ACMG/AMP strength |  | N (%) |  | N (%) |
| < 50 |  |  |  |  |  |  |  |  |  |  |  |  |  |
|  | HGSC |  | 630 (75.4) | 1.81 (1.66-1.98) | non-informative |  | 84 (71.8) | 1.73 (1.50-1.98) | non-informative |  | 365 (41.6) |  | 1,079 (59.0) |
|  | LGSC |  | 18 (2.2) | **0.18 (0.11-0.30)** | **Moderate Benign** |  | 6 (5.1) | **0.43 (0.19-0.96)** | **Supporting Benign** |  | 104 (11.9) |  | 128 (7.0) |
|  | Mucinous |  | 13 (1.6) | **0.11 (0.06-0.19)** | **Moderate Benign** |  | 6 (5.1) | **0.36 (0.16-0.80)** | **Supporting Benign** |  | 124 (14.1) |  | 143 (7.8) |
|  | Endometrioid |  | 113 (13.5) | 0.73 (0.58-0.91) | non-informative |  | 14 (12.0) | 0.64 (0.39-1.07) | non-informative |  | 163 (18.6) |  | 290 (15.8) |
|  | Clear cell |  | 10 (1.2) | **0.16 (0.08-0.31)** | **Moderate Benign** |  | 4 (3.4) | 0.46 (0.17-1.24) | Benign Supporting |  | 65 (7.4) |  | 79 (4.3) |
|  | 'Other' |  | 52 (6.2) | 0.97 (0.68-1.40) | non-informative |  | 3 (2.6) | 0.40 (0.13-1.26) | Benign Supporting |  | 56 (6.4) |  | 111 (6.1) |
|  | Total |  | 836 |  |  |  | 117 |  |  |  | 877 |  | 1,830 |
| ≥ 50 |  |  |  |  |  |  |  |  |  |  |  |  |  |
|  | HGSC |  | 653 (75.2) | 1.08 (1.03-1.14) | non-informative |  | 359 (74.5) | 1.07 (1.01-1.14) | non-informative |  | 1,510 (69.4) |  | 2,522 (71.5) |
|  | LGSC |  | 31 (3.6) | 0.84 (0.57-1.26) | non-informative |  | 16 (3.3) | 0.79 (0.47-1.32) | non-informative |  | 92 (4.2) |  | 139 (3.9) |
|  | Mucinous |  | 7 (0.8) | **0.21 (0.10-0.44)** | **Moderate Benign** |  | 8 (1.7) | **0.42 (0.21-0.87)** | **Supporting Benign** |  | 85 (3.9) |  | 100 (2.8) |
|  | Endometrioid |  | 91 (10.5) | 0.99 (0.79-1.25) | non-informative |  | 46 (9.5) | 0.90 (0.67-1.22) | non-informative |  | 230 (10.6) |  | 367 (10.4) |
|  | Clear cell |  | 20 (2.3) | **0.37 (0.23-0.59)** | **Supporting Benign** |  | 9 (1.9) | **0.30 (0.15-0.58)** | **Supporting Benign** |  | 136 (6.3) |  | 165 (4.7) |
|  | 'Other' |  | 66 (7.6) | 1.35 (1.01-1.80) | non-informative |  | 44 (9.1) | 1.61 (1.16-2.25) | non-informative |  | 123 (5.7) |  | 233 (6.6) |
|  | Total |  | 868 |  |  |  | 482 |  |  |  | 2,176 |  | 3,526 |
|  |  |  | 1,704 |  | |  | 691 |  |  |  | 3,053 |  | 5,356 |

N, Number of data points; LR, Likelihood ratio; CI, Confidence Interval; ACMG/AMP, American College of Medical Genetics/Association for Molecular Pathology; HGSC, High-grade serous carcinomas; LGSC, Low-grade serous carcinomas. The ‘other’ category denominates rare forms of ovarian cancer not belonging to any of the other subtypes, including tumours defined as: ‘other’ by data sources not specifying tumour histology; mixed-epithelial; carcinosarcomas; transitional cell (Brenner tumours); undifferentiated or poorly differentiated; squamous cell. In brackets, the histotype frequency for each group is provided.LR>1: Histotype association with pathogenic variant, Pathogenic evidence; LR<1: Prediction of non-carrier for pathogenic variant, Benign evidence. Evidence strength was measured based on Bayesian modelling of ACMG/AMP rules (see Materials and Methods); Supporting Benign (LR≥0.23-0.48), Moderate Benign (LR≥0.053-0.23), Supporting Pathogenic (LR≥2.08-4.30), non-informative (0.48≤LR≤2.08). LR estimates reaching informative ACMG/AMP strengths at a statistically significant CI (i.e., not spanning 1), are highlighted in bold.
